# Electrically Tunable Momentum Space Polarization Singularities in Liquid Crystal Microcavities

**DOI:** 10.1002/advs.202500060

**Published:** 2025-05-14

**Authors:** Przemysław Oliwa, Piotr Kapuściński, Maria Popławska, Marcin Muszyński, Mateusz Król, Przemysław Morawiak, Rafał Mazur, Wiktor Piecek, Przemysław Kula, Witold Bardyszewski, Barbara Piętka, Helgi Sigurðsson, Jacek Szczytko

**Affiliations:** ^1^ Institute of Experimental Physics Faculty of Physics University of Warsaw ul. Pasteura 5 Warsaw PL‐02‐093 Poland; ^2^ Institute of Applied Physics Military University of Technology ul. gen. Kaliskiego 2 Warsaw PL‐00‐908 Poland; ^3^ Institute of Chemistry Military University of Technology ul. gen. Kaliskiego 2 Warsaw PL‐00‐908 Poland; ^4^ Institute of Theoretical Physics Faculty of Physics University of Warsaw ul. Pasteura 5 Warsaw PL‐02‐093 Poland; ^5^ Science Institute University of Iceland Dunhagi 3 Reykjavik IS‐107 Iceland

**Keywords:** liquid crystal microcavity, momentum space polarization singularities, meron polarization texture, polarization optics, polarization vortex, spin‐orbit coupling of light

## Abstract

Momentum space polarization singularities of light appear as vectorial twists in the scattered and radiated far field patterns of exotic photonic structures. They relate to important concepts such as bound states in the continuum, spatiotemporal light steering, polarization Möbius strips, Berry curvature, and associated topological photonic phenomena. Polarization singularities, such as completely circularly polarized C‐points, are readily designed in real space through interference of differently polarized beams. In momentum space, they require instead sophisticated patterning of photonic crystal slabs of reduced symmetries in order to appear in the corresponding band structure with scarce in situ tunability. Here, it is shown that momentum space singularities can be generated and, importantly, electrically tuned in the band structure of a highly birefringent planar liquid crystal microcavity that retains many symmetries. The results agree with theoretical predictions and offer exciting possibilities for integration of momentum space polarization singularities in spinoptronic technologies.

## Graphical abstract

Using a highly birefringent material embedded in a liquid crystal microcavity, the Rashba‐Dresselhaus resonance is obtained. In this regime, polarization singularities, known as C‐points, are observed in momentum space. The positions of these singularities can be modified by applying an external voltage to the sample. Additionally, different types of merons (Bloch or Néel), which can also be controlled by external voltage, are associated with these C‐points.

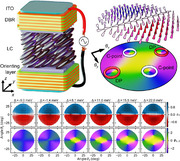

## 1 Introduction

Polarization singularities known as C‐points are twisted sources of highly circularly polarized light encircled by a quantized winding linear polarization vector in the electromagnetic field's parameter space.^[^ ^1^, ^2^ ^]^ More formally, they are phase‐singular points in the electromagnetic scalar field E·E where the orientation of the polarization ellipse is undefined. They possess topological charges intimately related to the field's geometric (Berry) phase^[^ ^3^ ^]^ and share strong similarities with electromagnetic textures known as optical skyrmions.^[^ ^4^ ^]^ C‐points can be readily generated in real space through superposition of beams with orthogonal photon spin‐ and orbital angular momentum^[^ ^1^ ^]^ utilizing e.g., q‐plates based on metasurfaces and/or liquid crystals technologies.^[^ ^5^, ^6^ ^]^ Only, recently has the interest of researchers been piqued towards polarization singularities in momentum (reciprocal) space^[^ ^7^, ^8^ ^]^ due to their implication for polarization generation,^[^ ^9^ ^]^ routing chiral emission in atomic monolayers,^[^ ^10^ ^]^ laser transmission through birefringent liquid crystal layers,^[^ ^11^ ^]^ polarimetry mapping of photonic Berry curvature,^[^ ^12^ ^]^ the far field radiation properties of patterned photonic structures hosting bound states in continuum (BICs)^[^ ^13^, ^14^, ^15^, ^16^, ^17^, ^18^ ^]^ or creation of polarization Möbius strips,^[^ ^19^ ^]^ etc.

Similar to scalar phase singularities like in optical vortex beams^[^ ^20^ ^]^ polarization singularities in multicomponent fields can be assigned a topological charge referred to as *Poincaré‐Hopf index* defined by a closed path integral in the field's parameter subspace,

(1) $$2w=\frac{1}{2\pi }\oint \nabla \phi_{\mu ,\nu }\cdot dl=1,2,3,\cdots$$

The factor 2 is conventional and relates C‐points to the winding numbers of optical merons or half‐charge skyrmions.^[^ ^4^ ^]^ Here, $\phi_{\mu ,\nu }=\arg\left(s_{\mu }+is_{\nu }\right)$ is the *Stokes phase* ^[^ ^3^ ^]^ containing the singularity and $s_{i}=E^{†}\sigma_{i}E/{\left|E\right|}^{2}$ are the Stokes parameters and $E={\left[E_{H},E_{V}\right]}^{T}$ is the paraxial cavity field in the linear polarization basis, and $\sigma =\left(\sigma_{z},\sigma_{x},\sigma_{y}\right)$ are the Pauli matrices. In contrast to typical scalar vortices, which have cores of zero intensity, the vectorial nature of the electromagnetic field permits C‐points to be finite and fully circularly polarized at their core (*s*_3_ = ±1) – hence the name – but of undefined linear polarization. There also exist nonradiative V‐points, which form points of zero total field intensity connected to BICs,^[^ ^16^ ^]^ and L‐lines, trajectories of pure linear polarization that separate regions of opposite handedness belonging to C‐points.^[^ ^21^ ^]^

With surging advancement in both beam steering and polarization manipulation within the nanophotonics sector through the use of miniaturized metasurfaces, photonic crystal slabs, and gratings, new opportunities have opened to develop optical technologies based on polarization singularities in the far field reciprocal space.^[^ ^16^, ^22^, ^23^ ^]^ They could become a disruptive force for classical or quantum information processing purposes, enhanced light‐matter interaction in optical cavities, optical trapping, chiral sensing and lasing, etc.^[^ ^1^, ^2^ ^]^ This development underpins a need in designing microscale optical systems that not only passively generate pre‐determined polarization singularities, but can also be flexibly tuned in situ to smoothly adjust C‐point characteristics and their number. So far, implementations of momentum space polarization singularities rely mostly on irreversibly patterned materials such as photonic,^[^ ^7^, ^13^ ^]^ polaritonic,^[^ ^24^, ^25^, ^26^ ^]^ or plasmonic^[^ ^8^, ^27^ ^]^ crystal slabs or heterostructures. Control over the singularity type and location in reciprocal space is achieved by reducing or breaking symmetries of the slab such going from fourfold (*C*_4*v*_) to twofold (*C*_2*v*_) rotational in‐plane symmetry,^[^ ^27^ ^]^ or the up‐down^[^ ^15^ ^]^ or in‐plane^[^ ^14^ ^]^ inversion symmetry. However, once the sample is created its properties are set and the singularities cannot be tuned or adjusted unless another sample with new parameters is created. This severely limits the application of momentum space polarization singularities in optical technologies and experiments requiring in situ tuning.

Here, we overcome this challenge, and demonstrate electrically tunable multiple half‐charge momentum space polarization singularities in the transmitted far field of an optical microcavity filled with uniaxial nematic liquid crystal as recently proposed.^[^ ^28^ ^]^ The singularities stem from the strong birefringence of the anisotropic liquid crystal microcavity (LCMC), which leads to effective photonic spin‐orbit coupling (SOC)^[^ ^29^ ^]^ and emergent optical activity, despite many cavity symmetries staying intact. We identify two types of singularities, non‐degenerate C‐points characterized by strong circular polarization, and degenerate diabolical points (DPs) that break into pairs of exceptional points (EPs) through polarization dependent losses.^[^ ^30^ ^]^

## 2 Results

### 2.1 Liquid Crystal Microcavity Sample

**Figure 1:**
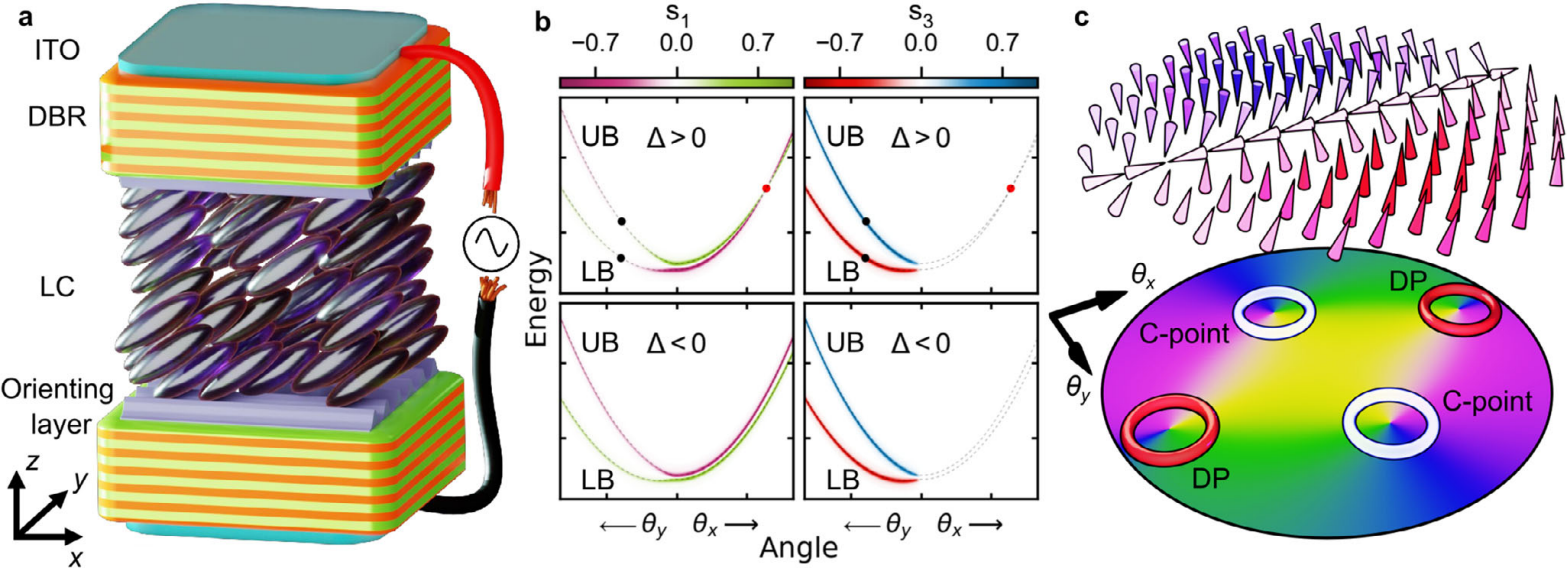
Liquid crystal microcavity device alongside the cavity photon dispersion and associated momentum space polarization singularities. a) Schematic figure of the electrically tunable LCMC device. b) Calculated polarization resolved cavity dispersion relation around the equal Rashba‐Dresselhaus SOC regime for two different Stokes parameters $s_{1,3}$ and detunings Δ. The black and red dots denote the location of C‐points and diabolical points, respectively. The UB and LB denote the upper and lower photonic branches, with energies $\hbar \omega_{+}$ and $\hbar \omega_{-}$, respectively, where $\omega_{+}\geq \omega_{-}$.

The studied LCMC is presented schematically in **Figure** **1**a. Additionally, in Figure S1 (Supporting Information) in ref. [31] we present the photograph of the sample. The cavity consists of a birefringent medium layer – uniaxial liquid crystal (LC) in a nematic phase – embedded between two parallel distributed Bragg reflectors (DBRs). Transparent ITO electrodes, covered by the DBRs, enable electrical control over the spatial orientation of the LC molecular director. This reorientation of the LC director within the *xz*‐plane modifies the projection of dielectric tensor on the *x*‐axis. Consequently, the linear polarization along the projection axis (here referred to as *horizontal* (*H*) polarization), changes energy, while the orthogonally polarized mode (*vertical* (*V*) polarization) remains unaffected (see Equation (S5), Supporting Information in ref. [31]). By adjusting the applied voltage, we can thus control the detuning between the *H*‐ and *V*‐polarized cavity modes, which align with the cavity's in‐plane directions, $\hat{x}$ and $\hat{y}$, respectively.

We work in the Rashba‐Dresselhaus (RD) regime^[^ ^32^, ^33^ ^]^ where degenerate *H* and *V* polarized cavity modes have staggered longitudinal parity. That is, H(m+1) and *V*(*m*), where *m* is the mode index, due to the extreme birefringence of the liquid crystal molecules. The cavity in‐plane momentum is defined as $k\equiv {\left[k_{x},k_{y}\right]}^{T}$ and is related to the angle of incidence of emitted light through $k=k_{0}\sin\left(\theta \right){\left[\cos\left(\varphi \right),\sin\left(\varphi \right)\right]}^{T}$, where *k*_0_ = 2π/λ, θ is the angle of incident wave and φ is the azimuthal in‐plane angle, so we defined $\theta ={\left[\theta_{x},\theta_{y}\right]}^{T}=\theta {\left[\cos\left(\varphi \right),\sin\left(\varphi \right)\right]}^{T}$. In what follows, data corresponding to reciprocal space (also referred to as *momentum* space) is plotted as a function of angle to relate directly to the experimental configuration.

### 2.2 Two‐Mode Photonic Hamiltonian

We start by establishing the presence of the polarization singularities in our RD LCMC system using two different theoretical approaches, both in good agreement with following experimental results. First, we calculate the LCMC dispersion relation using the Berreman and Schubert method^[^ ^34^, ^35^ ^]^ which has been widely successful including in our past experiments.^[^ ^30^, ^32^, ^33^, ^36^, ^37^, ^38^ ^]^ While highly accurate, the method is computationally time‐consuming and does not provide much physical insight into the observed polarization singularities.

For this reason, we also apply more recently developed approach based on optical cavity k·p perturbation theory,^[^ ^39^ ^]^ offering a simple coupled‐mode description in the form of a 2 × 2 Hamiltonian. This method provides a more transparent, quantum mechanics inspired, framework to analyze cavity photon SOC effects between two quasiresonant orthogonal linearly‐polarized LCMC modes without any phenomenological parameters, based on the Hamiltonian in the form:

(2) $$H=h_{0}\left(k\right)1_{2\times 2}+h\left(k\right)\cdot \sigma$$

The Hamiltonian is written in a rotated polarization basis to properly account for interactions between cavity modes.^[^ ^39^ ^]^ Here, $h_{i}\left(k\right)$ are in‐plane momentum‐dependent coefficients that are complex (rendering this Hamiltonian non‐Hermitian) due to the finite‐lifetime cavity photon states. We stress that the order of the Pauli matrices is $\sigma =\left(\sigma_{z},\sigma_{x},\sigma_{y}\right)$. The coefficients depend on the elements of the dielectric tensor^[^ ^31^ ^]^ and can be written in the parabolic approximation:

(3) $$h_{0}=\omega_{0}+i\Gamma_{0}+\frac{\hbar k_{x}^{2}}{2m_{x}}+\frac{\hbar k_{y}^{2}}{2m_{y}}$$

(4) $$h_{1}=\Delta +i\delta \Gamma +\delta_{x}k_{x}^{2}+\delta_{y}k_{y}^{2}$$

(5) $$h_{2}=0,$$

(6) $$h_{3}=-2\alpha k_{y}$$

here, ω0=Re(ωH(m+1)+ωV(m))/2 and Γ_0_ = Im(ω_*H*(*m* + 1)_ + ω_*V*(*m*)_)/2 are the mutual frequency and decay rate of the *H*, *V* cavity photons, and Δ = Re(ω_*H*(*m* + 1)_ − ω_*V*(*m*)_)/2 and δΓ = Im(ω_*H*(*m* + 1)_ − ω_*V*(*m*)_)/2 are their frequency and decay rate differences at normal incidence k=0. The remaining parameters linear and quadratic in k are detailed in the Supporting Information in ref. [31]

The eigenvalues and right eigenvectors of Hamiltonian in Equation (2) are:

(7) $$\omega_{\pm }=h_{0}\pm \sqrt{h_{1}^{2}+h_{3}^{2}}$$

(8) $$q_{\pm }=\left[\begin{array}{c} h_{1}\pm \sqrt{h_{1}^{2}+h_{3}^{2}}\\ ih_{3} \end{array}\right]$$

The spectrum of the LCMC is given by the eigenvalues (7) and we refer to ω_±_ as upper and lower frequency branches. The eigenstates (8) define the Stokes components of the cavity field which, upon transformation to the canonical cavity basis,^[^ ^40^ ^]^ are written as (not normalized):^[^ ^39^ ^]^

(9) $$\begin{array}{c} S_{i,\pm }\left(k\right)=\sum_{s,s^{\prime }}q_{\pm ,s}^{*}q_{\pm ,s^{\prime }}\left(\sigma_{i,ss^{\prime }}+\sum_{s^{\prime \prime }m^{\prime \prime }}R_{s^{\prime \prime }m^{\prime \prime },sm}^{*}\sigma_{i,s^{\prime \prime }s^{\prime }}+\sigma_{i,ss^{\prime \prime }}R_{s^{\prime \prime }m^{\prime \prime },s^{\prime }m^{\prime }}\right)\;\;\; \end{array}$$

where *s* and *s*′ denote vector elements of $q_{\pm }$, and $R_{sm,s^{\prime }m^{\prime }}$ represents an element of the rotation matrix R(k) detailed in the Supporting Informtion in ref. [31]. The *sm* and *s*′*m*′ elements denote the modes in the Hamiltonian Equation (2), so *sm* ∈ {*H*(*m* + 1), *V*(*m*)} and the same for *s*′*m*′. The *s*″*m*″ refers to all the states in the cavity that are different from the *sm* states. Equation (9) is cumbersome but, differently from previous two‐mode phenomenological models, it allows accurate ab initio calculation of the polarization field.

Having established the spectral Equation (7) and polarization measurables Equation (9) of our system, we can highlight the presence of polarization singularities by plotting the dispersion relation of the *s*_1_ and *s*_3_ Stokes parameters along *k*_*x*_ and *k*_*y*_ in Figure 1b for positive (Δ > 0) and negative (Δ < 0) detuning. We remind that *s*_1, 2, 3_ denote the degree of horizontal‐vertical linear polarization, diagonal‐antidiagonal linear polarization, and left‐ and right‐hand circular polarization of the bands, respectively.

As can be seen in Figure 1b, the bands are strongly circularly polarized along *k*_*y*_ direction (denoted by the blue‐red *s*_3_ colorscale) due to the Rashba‐Dresselhaus term [see Equation (6)]. The singularities can be located in the dispersion for Δ > 0 where the sign of *s*_1_ inverts between the bands. Non‐degenerate C‐points are marked with black dots and a degenerate DP with a red dot. In the former, the degree of linear polarization $\sqrt{s_{1}^{2}+s_{2}^{2}}$ goes to zero while the circular polarization |*s*_3_| = 1 is maximal. For the latter, the bands cross (in real energy) forming a so‐called DP^[^ ^27^ ^]^ in the Hermitian limit, which breaks into a pair of EPs in the non‐Hermitian case, bridged by a Fermi arc (not shown). In a negatively detuned cavity Δ < 0 (lower panels of Figure 1b) both C‐points and DPs disappear which we explain in the following.

Figure 1c illustrates these singularities more vividly in Stokes vector field and phase $\phi_{1,2}\left(k\right)$ of the upper branch. The C‐points are identifiable as lemon‐type *w* = 1/2 polarization singularities whereas the diabolical points as star‐type with opposite phase winding *w* = −1/2. The position of the C‐points for a given branch can be derived in the Hermitian limit (see Supporting Information in ref. [31]):

(10) $$k_{y,CP}=\pm \sqrt{\frac{\Delta }{\Sigma \left(m\right)\frac{4Lc\sqrt{m\left(m+1\right)}}{\pi^{3}\left(2m+1\right)}{\left(\frac{\varepsilon_{xz}}{\varepsilon_{zz}}\right)}^{2}-\delta_{y}}}$$

where *L* is the cavity thickness, *c* is the speed of light, $\varepsilon_{ij}$ are elements of the dielectric tensor, and Σ(*m*) describes correction from other cavity branches. Equation (10) explains why C‐points should vanish in negatively detuned microcavity. Although the sign of δ_*y*_ varies with θ, the value of Σ(m) remains strictly positive and large in the RD regime (see Supporting Information in ref. [31]), ensuring that the denominator is greater than zero. Consequently, within the presented model C‐points can only exist when Δ > 0.

Moreover, Equation (10) shows that, in the Hermitian limit, the two‐mode Hamiltonian – commonly used in microcavity studies^[^ ^41^, ^42^ ^]^ – predicts two C‐points per branch but incorrectly places them at the same wavevector positions across branches, contradicting transfer matrix simulations.^[^ ^28^ ^]^ Introducing losses to construct a non‐Hermitian model results in non‐orthogonal eigenvectors, leading to non‐orthogonal Stokes polarization parameters on the Poincaré sphere (see Section Non‐Hermitian Modification of two‐Mode Hamiltonian due to DBR Modes). This modification successfully separates the C‐point positions between branches.

For the DPs, it is important to stress that, due to non‐Hermiticity, they will separate into two pairs of EPs connected by Fermi arcs.^[^ ^30^, ^43^, ^44^, ^45^ ^]^ However, the EPs are so close to each other at this scale that they appear as half‐charge polarization singularities of star‐type *w* = −1/2 on opposite sides of the *k*_*x*_ axis. To better observe the structure of the EP pairs, we show zoomed in regions of the results in Figures S2 and S3 (Supporting Information) in ref. [31]. We note that an EP would exhibit pure circular polarization, like a C‐point, only if the cavity possesses mirror symmetry in the *x* − *y* plane.^[^ ^46^ ^]^ However, in this case, the cavity belongs to the *C*_2*h*_ symmetry point group with *C*_2_ axis along *y*‐axis, allowing for all Stokes polarization parameters to be non‐zero around the EPs.

An EP occurs when $h_{1}^{2}+h_{3}^{2}=0$, which for Δ ≫ δΓ yields following equation:

(11) $$k_{x,EP}=\pm \sqrt{-\frac{\Delta }{\delta_{x}}}\left(1+\frac{\delta_{y}\delta \Gamma^{2}}{8\alpha^{2}\Delta }\right)$$

(12) $$k_{y,EP}=\pm \frac{\delta \Gamma }{2\alpha }$$

Hence, we can observe four EPs located near the *k*_*x*_ axis. In the Hermitian limit δΓ → 0 we retrieve the location of the two DPs. Equation (11) provides a condition for the existence of these degeneracy points: Δ/δ_*x*_ < 0. Since δ_*x*_ < 0 for any rotation angle of the molecule θ, the points can only appear in the case of positive detuning Δ > 0. Because the anti‐hermitian part of our Hamiltonian is weak (but not negligible) we will—for simplicity—refer to the locality in momentum space hosting EP pairs as a DPs.

### 2.3 Experimental Results

**Figure 2:**
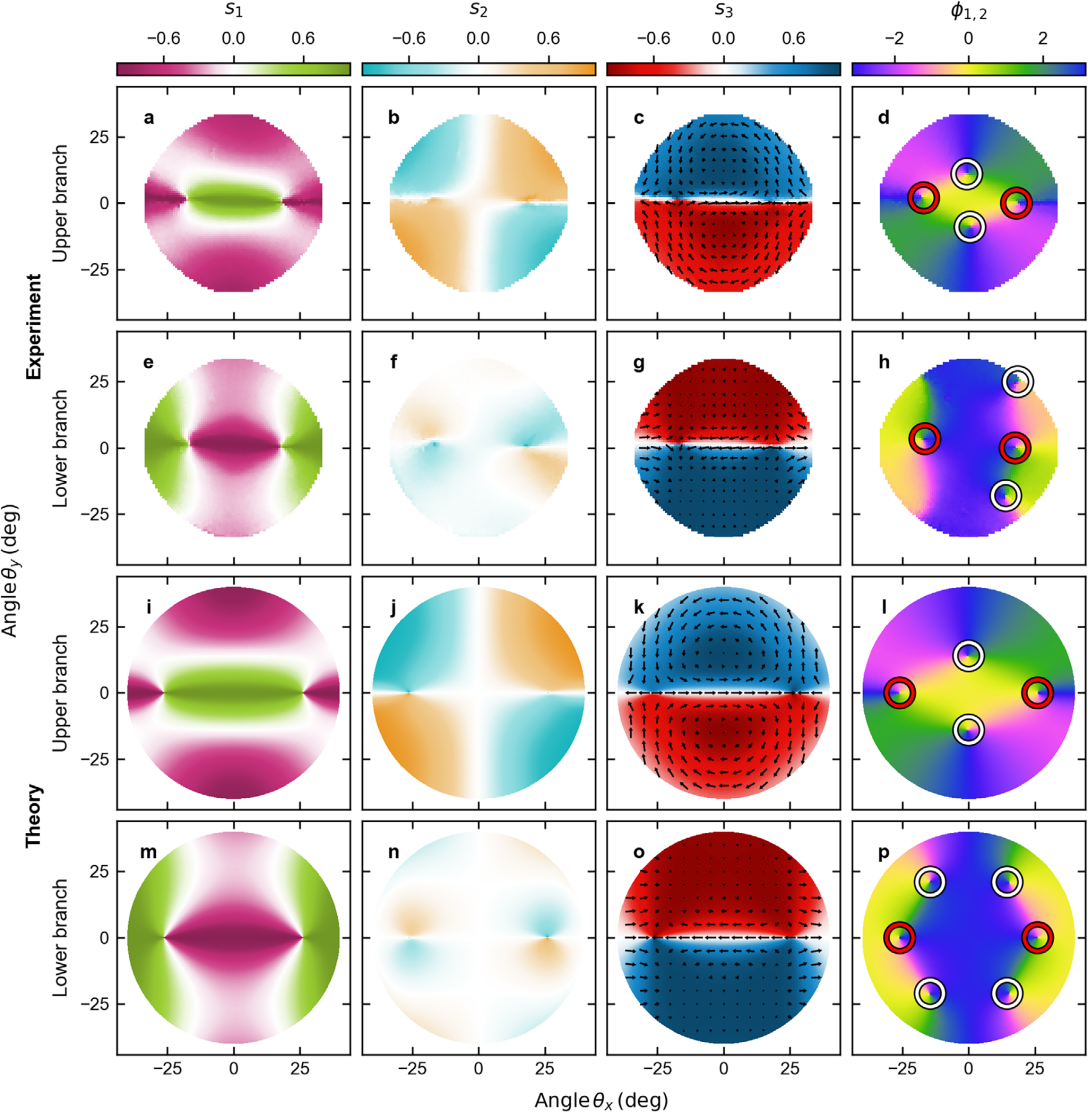
Stokes parameters and phase for positive detuning. a–h) Experimentally and i–p) theoretically obtained Stokes parameters (a, e, i, m) *s*_1_, (b, f, j, n), *s*_2_, **(**c, g, k, o) *s*_3_, and (d, h, l, p) Stokes phase $\phi_{1,2}=\arg\left(s_{1}+is_{2}\right)$ for (a–d, i–l) upper and (e–h, m–p) lower branch in the case of the positive detuning of 2ℏΔ=ℏRe(ωH(m+1)−ωV(m))=11.0meV. The black arrows on the $s_{3}$ map correspond to $s_{∥}={\left[s_{1},s_{2}\right]}^{T}$. The white and red circles on the Stokes phase $\phi_{1,2}$ maps mark the position of the C‐points and pair of EPs, respectively.

To experimentally verify the presence of polarization singularities in the LCMC dispersion, we performed angle‐resolved tomography of polarized white light transmission. We begin with Stokes parameter results for relatively large positive detuning (compared to the mode linewidth of 4 – 6 meV), 2ℏΔ = 11.0 meV, for the upper branch in **Figure** **2**a–d and the lower branch in Figure 2e–h. The overlaid arrows in Figure 2c,g represent the field's linear polarization, i.e., projection of the Stokes vector $s_{∥}={\left[s_{1},s_{2}\right]}^{T}$. Figure 2d,h show the Stokes phase $\phi_{1,2}=\arg\left(s_{1}+is_{2}\right)$.

In agreement with our two‐mode theory [see Equation (2)] and transfer matrix calculations, the upper branch reveals two lemon‐type *w* = 1/2 C‐points along the θ_*x*_ ≈ 0° direction and two star‐type *w* = −1/2 degenerate polarization singularities corresponding to DPs along the θ_*y*_ ≈ 0° direction, marked by white and red circles respectively in the Stokes phase map in Figure 2d,l. The direction of the linear polarization winds around these points resulting in finite curl of the vector field $s_{∥}$. As expected, near the C‐points, we observe a high degree of circular polarization $|s_{3}\left|\gg \right|s_{∥}|$. In the lower branch, our theory based on transfer matrix calculation reveals four C‐points located at $\theta \approx {\left[\pm 14^{\circ },\pm 21^{\circ }\right]}^{T}$ and two DPs at $\theta \approx {\left[\pm 25^{\circ },0\right]}^{T}$ (see Figure 2m–p). We did not observe two C‐points at $\theta \approx {\left[-14^{\circ },\pm 21^{\circ }\right]}^{T}$ experimentally, likely due to a combination of two factors. First, although the *s*_2_ parameter changes sign when crossing these C‐points, its absolute variation is small (compare Figure 2f,n), necessitating highly precise measurements. Second, the reduced signal intensity at negative θ_*x*_ values, caused by a slight tilt of the sample in the experimental setup with respect to the beam axis, increased the experimental uncertainty, making it difficult to capture the sign change of the *s*_2_ parameter at these C‐points. Nonetheless, the agreement between transfer matrix calculations and experiment is striking.

However, the second‐order non‐Hermitian Hamiltonian, fails to reproduce the four C‐points observed in the lower branch. As revealed by transfer matrix simulations, the second Stokes polarization parameter for the upper branch *s*_2, +_, changes sign only along the *k*_*x*_ = 0 $\left(\theta_{x}=0\right)$ and *k*_*y*_ = 0 $\left(\theta_{y}=0\right)$ directions. In contrast, *s*_2, −_ exhibits an additional sign change along a closed curve resembling an ellipse. Reproducing this feature requires complex higher‐order wavevector‐dependent terms, arising from the polarization‐dependent interactions with other cavity modes. While previous studies have explored higher‐order non‐Hermitian terms, these are typically proportional to $\sigma_{0}$,^[^ ^47^ ^]^ meaning they do not influence polarization patterns and thus cannot account for the observed behavior.

Our simulations indicate that the two additional C‐points arise from interactions with other cavity modes, primarily those confined within the DBRs. Figure S4 (Supporting Information) in ref. [31] presents Stokes phase $\phi_{1,2}\left(k\right)$ for various detuning values and cavity geometries (details in ref. [31]). The results for additional detuning values are presented in four supplementary movies (details in Section SIV of ref. [31]). Notably, these additional C‐points in the lower branch are absent only in cavities with DBRs featuring an inverted layer ordering and six or fewer layer pairs (see Figure S4ah‐am, Supporting Information ref. [31]). In all other cases – including the experimentally studied cavity (Figure S4aa‐af, Supporting Information in ref. [31]), a cavity with DBRs reduced to four layer pairs (Figure S4ao‐at, Supporting Information in ref. [31]), and a cavity with inverted DBR order and eight layer pairs (Figure S4av‐ba, Supporting Information in ref. [31]) – we observe two C‐points for negative detuning and four for positive detuning in the lower branch.

These results suggest that the occurrence and positioning of these additional C‐points are linked to interactions between cavity and DBR modes. A detailed discussion of this interaction can be found in Section SIV of ref. [31]. As outlined there, these interactions can be considered higher‐order wavevector terms in the two‐mode non‐Hermitian Hamiltonian.

**Figure 3:**
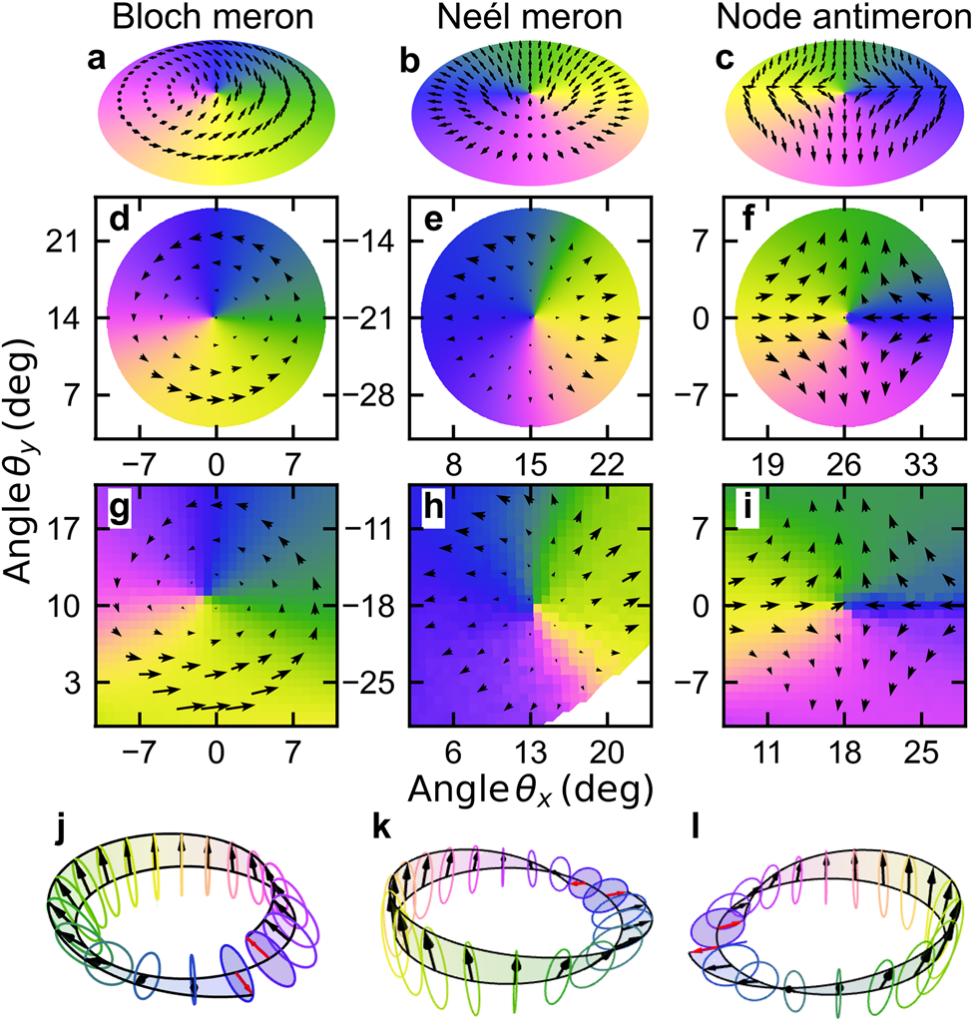
Distribution of Stokes parameters, phase and polarization ellipse for three different type of merons. a–c) Distribution of vector of Stokes polarization parameters s and Stokes phase for Bloch meron, Neél meron and Node anti‐meron in ideal case, respectively, (see Section SV of Supporting Information in ref. [31]). d–f) The same distribuiton obtained from Berreman and Schubert method and g‐i) for experimental data. j**‐**l) Möbius strips created by the polarization ellipse along a contour surrounding the polarization singularity. The arrows indicate the major semi‐axes of the ellipse, with the red arrow denoting the discontinuity in the major axis. The polarization ellipses are rotated by an angle of $\phi_{1,2}/2+\pi /2$ relative to the axis tangent to the circle.

The two‐mode non‐Hermitian and Hermitian Hamiltonians both predict that C‐points in each branch should be of the lemon type with the same helicities, based on skyrmionic considerations.^[^ ^4^ ^]^ However, experimental and fully numerical results reveal different helicities. Upper branch C‐points show characteristics similar to first‐order Bloch merons [see **Figure** **3**a,d,g], whereas the lower branch exhibits first‐order Néel merons [see Figure 3b,e,h]. The difference between these Bloch‐ and Néel‐meron textures can also be appreciated from the 2D Stokes divergence $\gamma_{1,2}=\partial_{k_{x}}s_{1}+\partial_{k_{y}}s_{2}$, where γ_1, 2_ ≈ 0 and ≠ 0 close to the Bloch‐ and Néel‐meron, respectively (see Figure S5 and S6, Supporting Information in ref. [31]). The difference between Bloch and Néel merons can be observed in the dispersion relation measured in the experiment. For a Bloch meron, the Stokes polarization parameters *s*_1_ and *s*_2_ change sign along the *k*_*y*_ (θ_*y*_) and *k*_*x*_ (θ_*x*_) directions, respectively (see Figure S7, Supporting Information in ref. [31]). In contrast, for a Néel meron, these parameters change sign along the *k*_*x*_ and *k*_*y*_ directions, respectively (see Figure S8, Supporting Information in ref. [31]). Additionally, we can change the C‐points from Bloch to Néel type by simply adjusting the detuning Δ which flips the meron helicity from 0 to π/2 (see Figures S5 and S6, Supporting Information in ref. [31]). Similar transformations between magnetic Néel skyrmion and Bloch skyrmionic bubbles have been observed in van der Waals ferromagnet Fe_3−δ_GeTe_2_.^[^ ^48^ ^]^ In those materials the meron type is determined by a sample thickness and iron concentration. In contrast, our momentum space optical cavity “meron” transformation is controlled in situ by tuning the external voltage. Additionally, the electric field (represented by the polarization ellipses) around an odd number of C‐points forms a Möbius strip,^[^ ^19^ ^]^ as illustrated in Figure 3j–l for the three types of merons present in the system. By implementing in‐situ changes to the type of meron (from Bloch to Néel and vice versa), we can modify the orientation of the Möbius strip in reciprocal space and, for a given polarization, determine the wavevector at which the discontinuity of the major semi‐axis of the polarization ellipse occurs (see the red arrows in Figure 3j–l).

At the DPs the half‐charge polarization singularities resemble anti‐merons but with undefined polarization at the center, hence we refer to it as a node anti‐meron [see Figure 3c,f,i]. These textures have opposite vorticity compared to the Bloch and Néel merons and cannot be smoothly changed since they come from band degeneracy.

We performed the same experiment and numerical modeling for negative detuning (Δ < 0), with the results presented in Figure S9 (Supporting Information) in ref. [31]. As predicted, under negative detuning, we observed only two C‐points in the lower branch, with a polarization distribution similar to a Bloch meron (see Figure S10, Supporting Information in ref. [31]). These data were collected for Sample B, which features a liquid crystal with lower birefringence and reduced scattering, resulting in slightly narrower mode linewidths while maintaining similar total intensities. This is crucial due to the partial overlap of modes at low detuning values.

**Figure 4:**
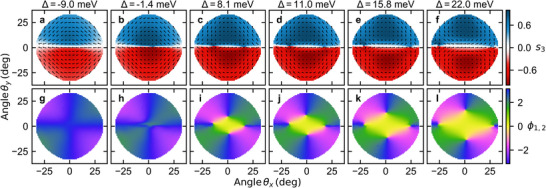
Tunability of the polarization singularities. a–f) Upper branch $s_{3}$ Stokes parameter for as a function of detuning with overlaid $s_{∥}={\left[s_{1},s_{2}\right]}^{T}$ as black arrows. g–i) Corresponding Stokes phase $\phi_{1,2}=\arg\left(s_{1}+is_{2}\right)$ showing changing position of the singularities in reciprocal space.

So far, we have demonstrated that in LCMC, we can alter both the number of C‐points and their helicity (Bloch or Néel meron‐like) by adjusting the detuning. Here, we further illustrate our ability to continuously tune the position of C‐points in momentum space. **Figure** **4** depicts the *s*_3_ Stokes polarization parameter, $s_{∥}$ vector, and the Stokes phase *ϕ*_1, 2_ for the upper branch for various detuning values in Sample A. The distribution of these parameters for the lower branch is presented in Figure S11 (Supporting Information) in ref. [31]. For negative detunings, no C‐points are observed. However, with positive detuning values, two C‐points appear, and importantly, their positions in reciprocal space can be smoothly adjusted by varying the detuning via external voltage control. Additionally, this tuning in reciprocal space can be easily observed in the dispersion relation for the *s*_1_ Stokes polarization parameter, as shown in Figure S12 (Supporting Information) in ref. [31].

## 3 Conclusion

We show that extreme birefringent liquid crystal microcavities can function as electrically tunable optical microscale devices for generation of multiple momentum‐space polarization singularities including purely circularly‐polarized points, C‐points with quantized winding of the associated linear polarization vector.

This offers a clear advantage over irreversibly nanopatterned photonic structures that must be carefully pre‐designed to achieve the desired output or transmission properties with little room for in situ adjustments. Given its electrical tunability, our device could prove useful to study in a controllable manner the evolution of geometric and dynamical phases,^[^ ^3^ ^]^ for optical‐based topological differential analysis,^[^ ^49^ ^]^ non‐diffracting space–time light bullets,^[^ ^50^ ^]^ and potentially exploring the role of optical skyrmions in quantum entanglement.^[^ ^51^ ^]^

Another exciting perspective of generating C‐points in microcavities, instead of photonic crystal slabs, is the high Q‐factor and small mode volumes of the microcavity that makes it convenient for lasing applications^[^ ^52^ ^]^ and to access the strong light‐matter coupling regime,^[^ ^53^ ^]^ even at room temperature.^[^ ^38^ ^]^ The latter leads to enhanced nonlinear effects in out‐of‐equilibrium condensation of exciton‐polaritons.^[^ ^54^, ^55^ ^]^ Furthermore, we have only studied the polarization structure of the transmitted cavity far field, which is mostly paraxial. A new venture into liquid crystal cavity optics would be to study electrically tunable 3D knotted C‐lines in a nonparaxial field, similar to those generated using spatial light modulator technologies.^[^ ^56^ ^]^

In addition, this work shows that the birefringent microcavity is an excellent platform for investigating non‐Hermitian photonic physics. The non‐orthogonality of the cavity eigenmodes (non‐Hermiticity) can be tuned by e.g., changing the number of pairs of layers in DBRs [see Section Non‐Hermitian Modification of 2‐Mode Hamiltonian due to DBR Modes in Experimental Section] and strongly depends on the angle of the incident wave. Therefore, future studies should consider the impact of higher‐order terms of the wavevector. Finally, this work shows that the description of the cavity is more complex than previously thought, making it a valuable platform for further developing Hamiltonians that describe non‐Hermitian physics.

## 4 Experimental Section

### Information About Sample

Sample A consisted of two DBRs composed of six pairs of layers TiO_2_/SiO_2_ with a central wavelength at 550 nm and SiO_2_ as a top layer. The dependence of refractive indices for these materials were presented in Figure S13 (Supporting Information) in ref. [31]. The DBRs were deposited on 25 nm ITO electrode layers on a 1.1 mm glass substrate with the flatness of λ/4 at 633 nm. The size of the substrate was about 25 mm × 20 mm. Antiparallel orienting layers (SE‐130, Nissan Chem., Japan) were deposited on both substrates using the spin‐coating method. The cavity was filled with a liquid crystal mixture LC2091* in the nematic phase by capillary action. The dependence of ordinary *n*_*o*_ and extraordinary *n*_*e*_ refractive index are presented in Figure S13 (Supporting Information) in ref. [31]. The thickness of the liquid crystal layer is approximately 1250 nm (all details are given in Section SIIIB1 of ref. [31]). The sample consists dye (P580 – Pyrromethene 580) in CHCl_3_ with mass concentration about 1%.

Sample B consisted of two DBRs composed of six pairs of TiO_2_/SiO_2_, with a central wavelength of 530 nm and SiO_2_ as a top layer. The dependence of refractive indices for these materials are presented in Figure S13 (Supporting Information) in ref. [31]. The ITO electrode, glass substrate, and orienting layers were the same as in sample A. Sample B was filled with the liquid crystal mixture LC1892* in the nematic phase through capillary action. It was assumed that the ordinary *n*_*o*_ and extraordinary *n*_*e*_ refractive index were independent of the wavelength and they were equal to *n*_*o*_ = 1.5190 and *n*_*e*_ = 1.7114. The thickness of the liquid crystal layer was approximately 2400 nm (all details are given in Section SIIIB2 of ref. [31])

### Data Acquisition and Analysis

The experimental results were obtained by polarization‐resolved tomography in transmission configuration. The broadband LED was used as a light source. The automated setup of the quarter‐wave and half‐wave plates with a fixed linear polarizer was used to achieve desired polarization state of the incident light. The polarized light was focused on a sample using a microscope objective with a numerical aperture of NA = 0.7. The electrodes of the sample were connected to an external voltage source with a square waveform signal of 1 kHz frequency. Transmitted light was collected with a microscope objective with NA = 0.75 and focused on a slit of a monochromator equipped with a CCD camera with a set of reciprocal space imaging lenses. By automatized move of the last lens coupled with an acquisition of the CCD camera, 3‐D maps of energy and momentum in $k_{x}\left(\theta_{x}\right)$ and $k_{y}\left(\theta_{y}\right)$ directions were reconstructed for six polarizations (horizontal (*H*), vertical (*V*), diagonal (*D*), antidiagonal (*A*), right‐hand circular (σ^+^), or left‐hand circular (σ^−^)) and for different voltage values, corresponding to certain values of detuning.

In our data analysis, two Lorentzian functions *f*_±, *i*_(λ, λ_±_, *A*_±, *i*_) = *A*_±, *i*_/(1 + ((λ − λ_±_)/γ_±_)^2^ were fitted to two orthogonally polarized spectra simultaneously, using the same width and peak position for both spectra while allowing the amplitudes to vary independently. This procedure yields two linewidths (γ_+_ and γ_−_) and energies (λ_+_ and λ_−_) (for the upper (+) and lower (−) branches) and four amplitudes (*A*_+, 1_ and *A*_+, 2_ for the upper and *A*_−, 1_ and *A*_−, 2_ lower branches in two orthogonal polarizations). The Stokes polarization parameters presented in Figures 2 and 3 were calculated as *S*_±_ = (*A*_±, 1_ − *A*_±, 2_)/(*A*_±, 1_ + *A*_±, 2_).

### Non‐Hermitian Modification of two‐Mode Hamiltonian due to DBR Modes

One issue requiring clarification was the reason behind the discrepancy between the experimental findings the simplified 2 × 2 Hamiltonian approach. At both positive and negative detunings, the experiment and Berreman‐and‐Schubert method reveal two additional C‐points in the lower branch that were not present in the 2 × 2 Hamiltonian solutions. Here, it was argued that this discrepancy arises due to the interaction of the cavity modes with the Bragg modes of the DBRs. Figures S14 and S15 (Supporting Information) in ref. [31] show the Stokes polarization parameters for the two branches calculated using the Berreman method for the photonic structure based on Sample A, but with four and eight pairs of layers in the DBRs, respectively. As shown in these figures, the polarization pattern for the lower branch strongly depends on the number of pairs of layers in DBRs. For the Hermitian 2 × 2 Hamiltonian matrix H written in the linear basis, the normalized eigenvectors $q_{\pm }$ (+ and − denotes the upper and lower branch, respectively) were orthogonal, so $\langle q_{\pm }|q_{\pm }\rangle =\delta_{\pm ,\pm }$, where δ_*ij*_ denotes the Kronecker delta. The normalized Stokes polarization parameters defined as $s_{i,\pm }=q^{†}\sigma_{i}q$, where σ is the same as in Equation (2), are orthogonal on the Poincaré sphere, which means that *s*_*i*, +_ = −*s*_*i*, −_. For a Hermitian system $\langle s_{+}|s_{-}\rangle =-1$, where $s_{\pm }={\left[s_{1,\pm },s_{2,\pm },s_{3,\pm }\right]}^{T}$. The numerical results show that with the increasing number of layers in DBRs, an increase in the non‐orthogonality in the system was observed, defined as $\chi =\left(\langle s_{+}|s_{-}\rangle +1\right)/2$, so for fully orthogonal states χ = 0, while for a fully non‐orthogonal system χ = 1. The increasing number of layers in the DBRs narrows the stopband, bringing the Bragg modes closer to the cavity modes. Consequently, this proximity enhanced their interaction, resulting in an increase in χ and a greater impact of non‐Hermiticity in the system under consideration. Figures S16 and Figure S17 (Supporting Information) in ref. [31] illustrate the values of χ for four, six, and eight pairs of layers for sample A and sample B, with the remaining parameters consistent with those in Figure 2 and Figure S9 (Supporting Information) in ref. [31], respectively.

The findings underscore the limitations of the 2 × 2 Hamiltonian in describing LCMCs and highlight several important considerations for accurately describing polarization patterns in liquid crystal microcavities. First, higher‐order expressions, including up to fourth order or more, may be crucial. The fourth order term in the non‐Hermitian Hamiltonian was crucial for obtaining two and four C‐points in the upper and lower branches, respectively. However, this fourth order term was sometimes insufficient to accurately reproduce the distribution of the imaginary part of the energy for the two branches, requiring higher‐order terms for proper modelling. For example, in a recent work^[^ ^47^ ^]^ authors include higher‐order wavevector terms (up to fourth order) but only in the imaginary component (which accounts for system losses). Second, a non‐Hermitian Hamiltonian matrix was necessary to describe phenomena like EPs^[^ ^30^, ^45^ ^]^ and non‐orthogonal states on the Poincaré sphere effectively. Third, the interaction between cavity modes and Bragg modes within distributed Bragg reflectors (DBRs) could significantly impact results, as proved by this work. Therefore, the 2 × 2 Hamiltonian should account for this interaction, possibly through perturbation theory or similar methods.

Additionally, the study highlights the necessity for a comprehensive approach to developing a suitable 2 × 2 matrix that incorporates all the aforementioned components, rather than proposing Hamiltonians without proper derivation, as was often the case. In a recent paper,^[^ ^39^ ^]^ a method was introduced for deriving a general formula for the 2 × 2 non‐Hermitian Hamiltonian for a birefringent cavity. However, this approach proves inadequate in this context because the Hamiltonian derived did not consider higher‐order terms in the wavevector or the influence of the DBR modes, as analysis was confined to cavities resembling Fabry‐Pérot resonators. Future work will focus on developing a model that accounts for these interactions.

## Conflict of Interest

The authors declare no conflict of interest.

## Supporting information

Supporting Information

Supplemental Movie S1

Supplemental Movie S2

Supplemental Movie S3

Supplemental Movie S4

## Data Availability

The data that support the findings of this study are openly available in Dane Badawcze UW Repozytorium at https://doi.org/10.58132/XCJJ6Z, reference number 58132.
