# Supplementary material for: Electrically Tunable Momentum Space Polarization Singularities in Liquid Crystal Microcavities
Source: Adv Sci (Weinh). 2025 May 14;12(23):2500060. doi: 10.1002/advs.202500060 (PMC12199379; doi:10.1002/advs.202500060)
Supplement: Supplementary file 1 — Supporting Information [file ADVS-12-2500060-s001.pdf]

ADVANCED SCIENCE
Open Access

Supporting Information

for *Adv. Sci.*, DOI 10.1002/advs.202500060

Electrically Tunable Momentum Space Polarization Singularities in Liquid Crystal Microcavities

Przemysław Oliwa, Piotr Kapuściński, Maria Popławska, Marcin Muszyński, Mateusz Król, Przemysław Morawiak, Rafał Mazur, Wiktor Piecek, Przemysław Kula, Witold Bardyszewski, Barbara Piętka, Helgi Sigurðsson and Jacek Szczytko**

Supporting Information – Electrically tunable momentum space polarization singularities in liquid crystal microcavities

Przemysław Oliwa,[1] Piotr Kapuściński,[1] Maria Popławska,[1] Marcin Muszyński,[1] Mateusz Król,[1] Przemysław Morawiak,[2] Rafał Mazur,[2] Wiktor Piecek,[2] Przemysław Kula,[3] Witold Bardyszewski,[4] Barbara Piętka,[1] Helgi Sigurðsson,[1,5,*] and Jacek Szczytko[1,†]

[1]*Institute of Experimental Physics, Faculty of Physics, University of Warsaw, ul. Pasteura 5, PL-02-093 Warsaw, Poland*
[2]*Institue of Applied Physics, Military University of Technology, ul. gen. Kaliskiego 2, PL-00-908 Warsaw, Poland*
[3]*Institue of Chemistry, Military University of Technology, ul. gen. Kaliskiego 2, PL-00-908 Warsaw, Poland*
[4]*Institute of Theoretical Physics, Faculty of Physics, University of Warsaw, ul. Pasteura 5, PL-02-093 Warsaw, Poland*
[5]*Science Institute, University of Iceland, Dunhagi 3, IS-107, Reykjavik, Iceland*

SI. INFORMATION ABOUT SAMPLE

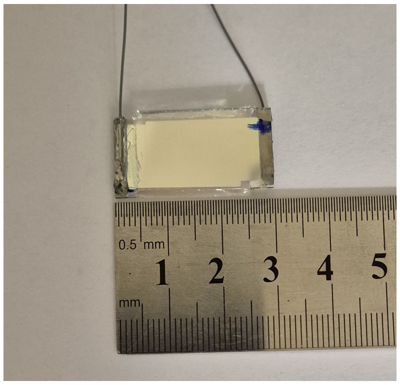

FIG. S1. The photo of typical liquid crystal microcavity.

Figure S1 shows a photograph of a typical liquid crystal microcavity used in the experiment. In this case, the image features Sample B. The standard size of such a cell is approximately 30 mm × 20 mm. The total thickness of the sample is slightly greater than 2 mm.

SII. 2-MODE HAMILTONIAN

A. Parameters of 2-mode Hamiltonian

In each equation, we used the following notation. The subscripts $s = X$ and $s = Y$ denote the horizontally and vertically polarized cavity mode, respectively. The subscripts $X\left(m\right)$ and $Y\left(m\right)$ denote the m-th horizontally and vertically polarized mode, respectively. The value of parameters of 2×2 Hamiltonian presented in Eq. (2) are equal to:

$$\omega_0 = \mathrm{Re}\left(\frac{\overset{o}{\omega}_{X(m+1)} + \overset{o}{\omega}_{Y(m)}}{2}\right) \tag{S1a}$$

$$\Gamma_0 = \mathrm{Im}\left(\frac{\overset{o}{\omega}_{X(m+1)} + \overset{o}{\omega}_{Y(m)}}{2}\right) \tag{S1b}$$

$$\Delta = \mathrm{Re}\left(\frac{\overset{o}{\omega}_{X(m+1)} - \overset{o}{\omega}_{Y(m)}}{2}\right) \tag{S1c}$$

$$\delta\Gamma = \mathrm{Im}\left(\frac{\overset{o}{\omega}_{X(m+1)} - \overset{o}{\omega}_{Y(m)}}{2}\right) \tag{S1d}$$

The $\overset{o}{\omega}_{sm}$ denote the resonant frequency for s polarized mode with m-th mode number, equal to [1]:

$$\overset{o}{\omega}_{sm} = \frac{c m_s \pi}{n_s L}\left(1 - \frac{1}{L\zeta_s} + \frac{1}{L^2\zeta_s^2} - \frac{i n_a m_s \pi}{2L^2\zeta_s^2 n_s}\right) \tag{S2}$$

where ζ_s denote the "strength of δ-mirrors" for s polarization, c is the speed of light, L is the cavity thickness, n_s is the cavity refractive index, and $n_a = 1$ is the outside refractive index. As mentioned in the manuscript, the liquid crystal molecules rotate along the y-axis when an external voltage is applied. In the initial state (without external voltage), the long axis of the molecule (the axis with the extraordinary refractive index, n_e) is aligned in the x-direction. Therefore, the dielectric tensor for this case is given by:

$$\varepsilon_d = \mathrm{diag}\left(n_e^2, n_o^2, n_o^2\right). \tag{S3}$$

The molecules rotate along the y-axis under an external voltage, so the dielectric tensor for any applied voltage is given by:

$$\varepsilon\left(\theta\right) = \mathbf{R}_y\left(\theta\right)\varepsilon_d\mathbf{R}_y^{\mathrm{T}}\left(\theta\right) = \begin{bmatrix} \varepsilon_{xx}\left(\theta\right) & 0 & \varepsilon_{xz}\left(\theta\right) \\ 0 & n_o^2 & 0 \\ \varepsilon_{zx}\left(\theta\right) & 0 & \varepsilon_{zz}\left(\theta\right) \end{bmatrix} \tag{S4a}$$

* Helgi.Sigurdsson@fuw.edu.pl
† Jacek.Szczytko@fuw.edu.pl

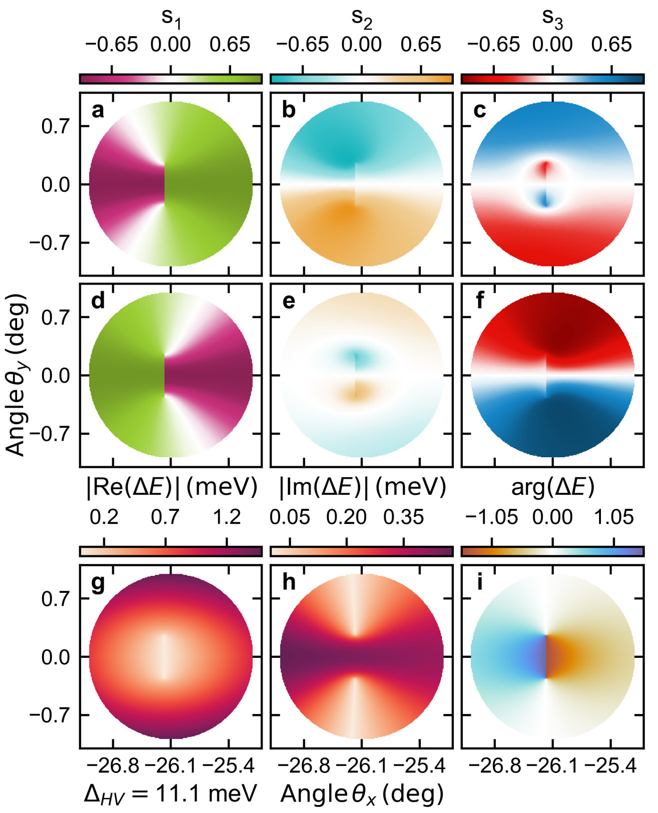

FIG. S2. Polarization patterns and difference between energies for the two cavity photon branches close to the EPs, corresponding to our analysis in Section II.B in the main manuscript. **a-c** and **d-f** present polarization patterns for upper and lower branches, respectively. **g** and **h** presents the absolute value of the real and imaginary part difference between energies for two branches, respectively. **i** presents the argument of complex number – difference between energies for both branches. The Δ_{HV} denote the difference between energies for two modes for perpendicular incident wave.

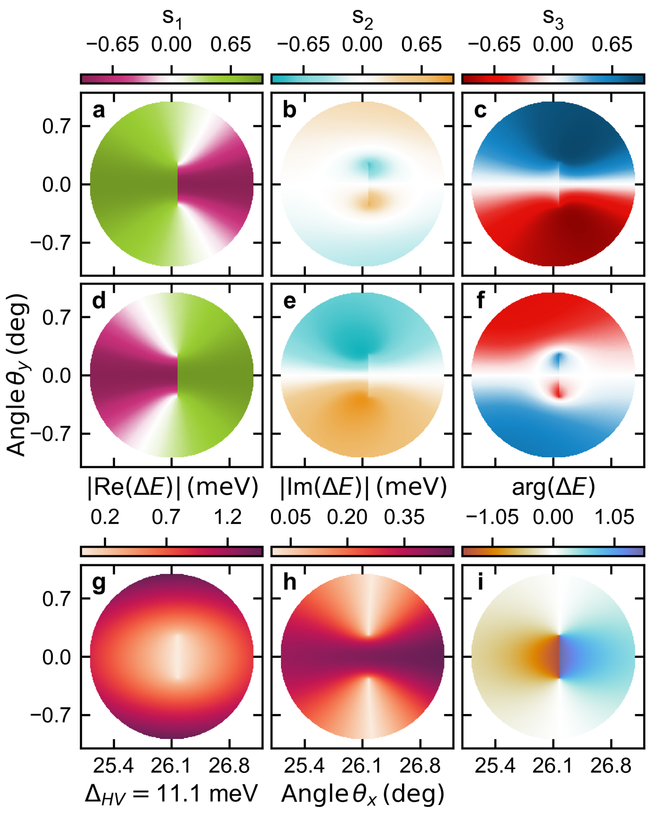

FIG. S3. Polarization patterns and difference between energies for the two cavity photon branches close to the EPs, corresponding to our analysis in Section II.B in the main manuscript. **a-c** and **d-f** present polarization patterns for upper and lower branches, respectively. **g** and **h** presents the absolute value of the real and imaginary part difference between energies for two branches, respectively. **i** presents the argument of complex number – difference between energies for both branches. The Δ_{HV} denote the difference between energies for two modes for perpendicular incident wave.

$$\varepsilon_{xx}(\theta) = n_e^2 \cos^2(\theta) + n_o^2 \sin^2(\theta) \tag{S4b}$$

$$\varepsilon_{zz}(\theta) = n_e^2 \sin^2(\theta) + n_o^2 \cos^2(\theta) \tag{S4c}$$

$$\varepsilon_{xz}(\theta) = \varepsilon_{zx}(\theta) = \left(n_e^2 - n_o^2\right) \sin(\theta) \cos(\theta) \tag{S4d}$$

where θ is a nonlinear function of the external voltage applied to the sample and $\mathbf{R}_y(\theta)$ is a standard rotation matrix along y-axis. The effective refractive indices for horizontally and vertically polarized modes are given by the elements of the dielectric tensor $\varepsilon(\theta)$ and are expressed as:

$$n_x = \sqrt{\varepsilon_{xx}(\theta) - \frac{\varepsilon_{xz}(\theta)\,\varepsilon_{zx}(\theta)}{\varepsilon_{zz}(\theta)}} = \\ = \frac{n_e n_o}{\sqrt{n_e^2 \sin(\theta) + n_o^2 \cos^2(\theta)}} \tag{S5a}$$

$$n_y = \sqrt{\varepsilon_{yy}(\theta) - \frac{\varepsilon_{yz}(\theta)\,\varepsilon_{zy}(\theta)}{\varepsilon_{zz}(\theta)}} = n_o \tag{S5b}$$

thus, the refractive index in the x-direction (horizontally polarized mode) can smoothly transition from n_e to n_o by applying an external voltage, while the refractive index in the y-direction (vertically polarized mode) remains constant throughout this process. This means that the energy of the horizontally polarized mode can be tuned, while the energy of the orthogonal mode remains constant [see Eq. (S2)].

The terms quadratic in transverse wave vector in Eq. (3) in the main text have the following form:

$$\frac{1}{m_x} = \frac{1}{2m_0}\left(\frac{a_x}{\eta} + b_x\eta\right) \tag{S6a}$$

$$\frac{1}{m_y} = \frac{1}{2m_0}\left(\frac{a_y}{\eta} + b_y\eta\right) \tag{S6b}$$

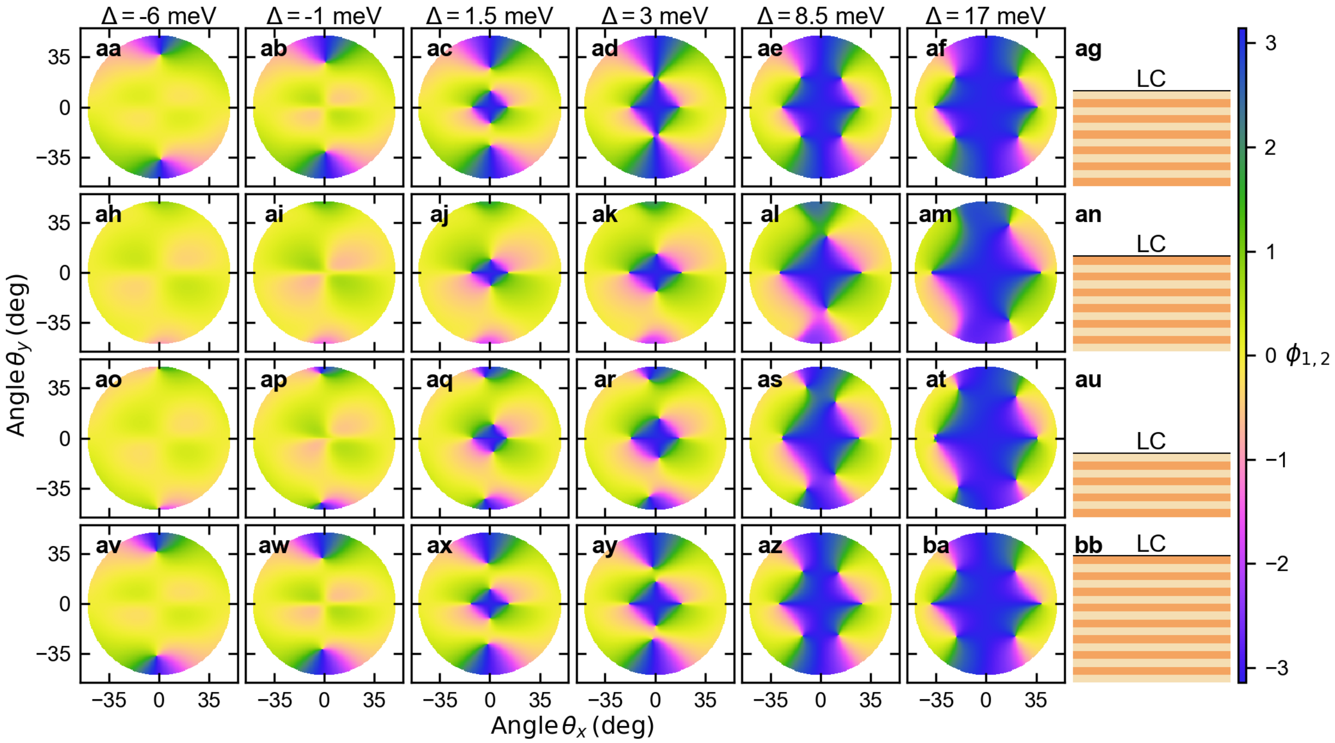

FIG. S4. Stokes phase ϕ_{12} for different values of detuning and different cavity types, along with a schematic representation of the layer arrangement in the DBRs. The first row (**aa-ag**) presents the Stokes phase (**aa-af**) and a schematic of the DBR layer distribution (**ag**), where the DBRs consist of six pairs of layers, with the terminating layer being a low-refractive-index layer. This cavity configuration matches the one used in the experiment. The second row (**ah-an**) shows the Stokes phase and a schematic of the sample, similar to the first row but with an inverted ordering of the DBR layers (see panel **an**). The third row (**ao-au**) presents the same type of information as the previous rows, but for a cavity with four pairs of layers arranged in the same order as in the first case (see panel **au**). The fourth row (**av-bb**) provides the same information for a cavity with eight pairs of layers, but with an inverted layer ordering compared to the first case (see panel **bb**).

$$\delta_x = \frac{\hbar}{4m_0}\left(\frac{a_x}{\eta} - b_x\eta\right) \tag{S6c}$$

$$\delta_y = \frac{\hbar}{4m_0}\left(\frac{a_y}{\eta} - b_y\eta\right) \tag{S6d}$$

The masses m_x and m_y describe the mean value of the curvature of two parabolas for two orthogonally polarized modes. The δ_x and δ_y describe the difference between these curvatures, which occur due to the presence of $TE-TM$ splitting, which is caused by strong birefringent of the medium inside the cavity. The other parameters are explained below. In the considered case, we also have one term linear in wave vector, whose coefficient is equal to:

$$\alpha = -\frac{\hbar}{4m_0}\frac{\varepsilon_{xz}}{n_X n_Y \varepsilon_{zz}} Q_{X(m+1),Y(m)}. \tag{S7}$$

This is the so-called Rashba-Dresselhaus coefficient [2]. The refractive indices for each polarization are $n_s = \sqrt{\tilde{\varepsilon}_{ss}}$, where $\tilde{\varepsilon}_{ij} = \varepsilon_{ij} - \varepsilon_{iz}\varepsilon_{zj}/\varepsilon_{zz}$ and ε_{ij} denotes the element of dielectric tensor. The $Q_{sm,s',m'}$ describe the overlap between a mode and the derivative of another mode along the optical axis of the cavity:

$$Q_{sm,s'm'} = \frac{n_s n_{s'}}{c^2}\int_0^L \left(\boldsymbol{E}_{sm}(z)\right)^T \partial_z \boldsymbol{E}_{s'm'}(z)\, \mathrm{d}z \approx$$
$$\approx \begin{cases} 0 & \text{if (1)} \\ \frac{4mm'}{L(m^2-(m')^2)} + \frac{8(m^3\Delta_{s'm'}-(m')^3\Delta_{sm})}{\pi(m^2-(m')^2)^2} & \text{if (2)} \end{cases} \tag{S8}$$

where (1) applies when $m+m'$ is even, and (2) applies otherwise. The rest mass of photon in a cavity m_0 and the normalization η are equal to:

$$m_0 = \frac{\hbar\sqrt{\overset{o}{\omega}_{X(m+1)}\overset{o}{\omega}_{Y(m)}}}{c^2} \tag{S9a}$$

$$\eta = \sqrt{\frac{\overset{o}{\omega}_{X(m+1)}}{\overset{o}{\omega}_{Y(m)}}} \tag{S9b}$$

The a_x, a_y, b_x and b_y parameters are defined by the elements of $\boldsymbol{\varepsilon}$ matrix, which describes the material inside

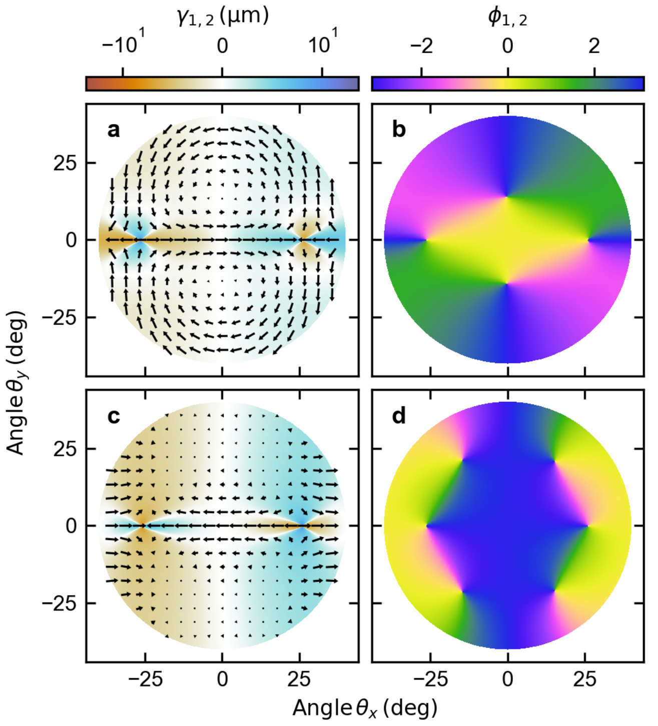

FIG. S5. Two dimensional divergence and Stokes phase for $\Delta = 11.1\,\mathrm{meV}$ for Sample A ($\theta = 31.4°$). **a** and **c** two dimensional divergence $\gamma_{1,2}$ for upper **a** and lower **c** branch, respectively. The arrows denote the $\boldsymbol{s}_{\parallel}$ vector. **b** and **d** Stokes phase for upper and lower branch, respectively.

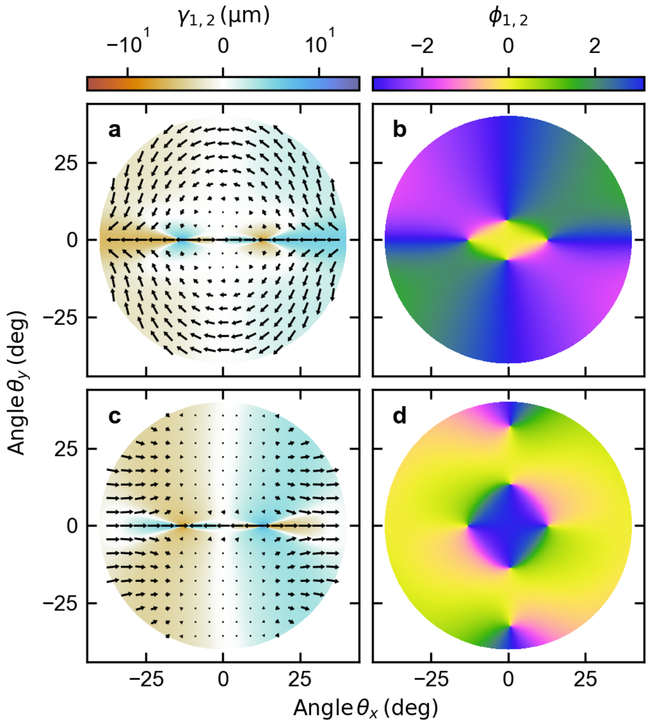

FIG. S6. Two dimensional divergence and Stokes phase for $\Delta = 2.4\,\mathrm{meV}$ for Sample A ($\theta = 29.5°$). **a** and **c** two dimensional divergence $\gamma_{1,2}$ for upper **a** and lower **c** branch, respectively. The arrows denote the $\boldsymbol{s}_{\parallel}$ vector. **b** and **d** Stokes phase for upper and lower branch, respectively.

the cavity:

$$a_x = \frac{1}{\tilde{\varepsilon}_{xx}}\left(\frac{\varepsilon_{xx}}{\varepsilon_{zz}} + 4\frac{\varepsilon_{xz}^2}{\varepsilon_{zz}} f_{X(m+1),X(m+1)}\left(X\right)\right) \tag{S10a}$$

$$b_x = \frac{1}{\varepsilon_{yy}} \tag{S10b}$$

$$a_y = \frac{1}{\tilde{\varepsilon}_{xx}}\left(1 + \frac{\varepsilon_{xz}^2}{\varepsilon_{zz}^2}\left(1 + f_{X(m+1),X(m+1)}\left(Y\right)\right)\right) \tag{S10c}$$

$$b_y = \frac{1}{\varepsilon_{yy}}\left(\frac{\varepsilon_{yy}}{\varepsilon_{zz}} + \frac{\varepsilon_{xz}^2}{\varepsilon_{zz}^2} f_{Y(m),Y(m)}(X)\right) \tag{S10d}$$

where the $f_{sm,s'm'}(s'')$ describes the impact from the other states present in the system, which is taken into account by perturbation theory. The first order correction are the terms quadratic in the wave vector and it is equal to:

$$f_{sm,s'm'}\left(s''\right) = -{\sum_{m''}}' \frac{c^2 Q_{sm,s''m''} Q_{s''m'',s'm'}}{4\overset{o}{\omega}_{s''m''} n_{s''}^2} \times \left(\frac{1}{\overset{o}{\omega}_{sm} - \overset{o}{\omega}_{s''m''}} + \frac{1}{\overset{o}{\omega}_{s'm'} - \overset{o}{\omega}_{s''m''}}\right) \tag{S11}$$

The sum $\sum_{m''}{}'$ is over all states which are different than the set of states belonging to 2-mode Hamiltonian, which in this case are equal to $X\left(m+1\right)$ and $Y\left(m\right)$. Due to taking into account the impact from other states, the basis of the non-Hermitian Hamiltonian in Eq. (2) from the main article is different than standard one in which one mode is horizontally polarized and the second is vertically polarized. In this case, the relation between the electric field in rotated basis $\boldsymbol{E}'_{sm}\left(z\right)$ and in standard linear basis $\boldsymbol{E}_{sm}\left(z\right)$ is following:

$$\boldsymbol{E}'_{sm}\left(z\right) = {\sum_{s'm'}}' \left(\exp\left(\mathbf{R}\right)\right)_{s'm'} \boldsymbol{E}_{s'm'} \tag{S12}$$

where the summation is over all states different than the states belonging to the 2-mode Hamiltonian. The rotation operator $\mathbf{R}$ is defined as:

$$\mathbf{R}_{sm,s'm'} = \begin{cases} -\frac{\mathcal{H}^{(1)}_{sm,s'm'}}{\overset{o}{\omega}_{sm} - \overset{o}{\omega}_{s'm'}} & \text{if } (1) \\ 0 & \text{if } (2) \end{cases} \tag{S13}$$

where (1) denotes the case in which $\boldsymbol{E}_{sm}$ and $\boldsymbol{E}_{s'm'}$ belong to different subsets of the basis set and (2) denotes the opposite case. The $\mathcal{H}^{(1)}_{sm,s'm'}$ defines the linear term in $\boldsymbol{k}$ in multimode Hamiltonian in [1], so in this case it is equal to:

$$\mathcal{H}^{(1)}_{sm,s'm'} = \chi_{sm,s'm'} \mathbf{A}_{ss'} Q_{sm,s'm'} \tag{S14}$$

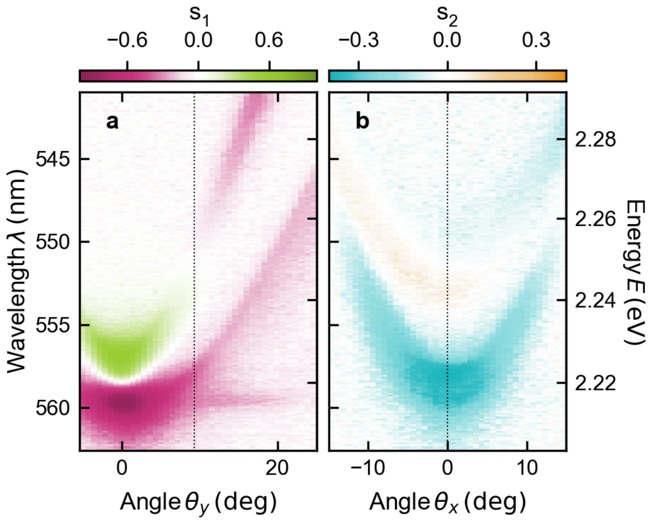

FIG. S7. The Stokes polarization parameters. **a** s_1 Stokes polarization parameter along k_y direction. **b** s_2 Stokes polarization parameter along k_x direction. The dotted black line denotes the value of k_y or k_x for which the other plot was done.

where $\chi_{sm,s'm'} = c^2/2n_s n_{s'}\sqrt{\overset{o}{\omega}_{sm}\overset{o}{\omega}_{s'm'}}$ and $\mathbf{A}$ is equal to:

$$\mathbf{A} = -i\frac{\varepsilon_{xz}}{\varepsilon_{zz}}\begin{bmatrix} 2k_x & k_y \\ k_y & 0 \end{bmatrix} \tag{S15}$$

B. Derivation of position of C-points

The Hamiltonian presented in the main text in Eq. (2) has the following two eigenvectors:

$$\boldsymbol{q}_{\pm} = \begin{bmatrix} h_1 \pm \sqrt{h_1^2 + h_3^2} \\ ih_3 \end{bmatrix} \tag{S16}$$

where h_0, h_1 and h_3, as shown in Eq. (3), are equal to:

$$h_0 = \omega_0 + i\Gamma_0 + \frac{\hbar k_x^2}{2m_x} + \frac{\hbar k_y^2}{2m_y}, \tag{S17a}$$

$$h_1 = \Delta + i\delta\Gamma + \delta_x k_x^2 + \delta_y k_y^2, \tag{S17b}$$

$$h_3 = -2\alpha k_y. \tag{S17c}$$

In our analysis, $\mathrm{Im}\,(h_1) \neq 0$ and $\mathrm{Im}\,(h_3) \neq 0$, which results in a complicated expression for $\boldsymbol{q}_{\pm}^{\dagger}$. However, when deriving the positions of the C-points, we perform the calculations in the Hermitian limit ($\zeta_i \to \pm\infty$), where $\mathrm{Im}\,(h_1) = 0$ and $\mathrm{Im}\,(h_3) = 0$, and $\boldsymbol{q}^{\dagger}$ is simply given by:

$$\boldsymbol{q}_{\pm}^{\dagger} = \left[h_1 \pm \sqrt{h_1^2 + h_3^2}, -ih_3\right], \tag{S18}$$

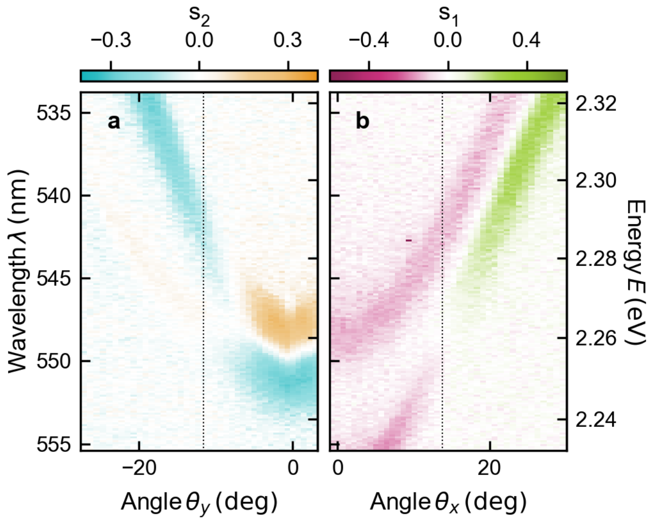

FIG. S8. The Stokes polarization parameters**a** s_2 Stokes polarization parameter along k_y direction. **b** s_1 Stokes polarization parameter along k_x direction. The dotted black line denotes the value of k_y or k_x for which the other plot was done.

which is just the conjugate transpose of Eq. (S16). In the Hermitian limit, as $\Delta_{sm} \to 0$, the formulas for ω_{sm}, $\chi_{sm,s'm'}$, and $Q_{sm,s'm'}$ simplify to the followings:

$$\omega_{sm} = \frac{cm\pi}{Ln_s} \tag{S19a}$$

$$\chi_{sm,s'm'} = \frac{cL}{2\pi\sqrt{mm'n_s n_{s'}}} \tag{S19b}$$

$$Q_{sm,s'm'} = \begin{cases} 0 & \text{if } m+m' \text{ even,} \\ \frac{4mm'}{L(m^2-(m')^2)} & \text{if } m+m' \text{ odd} \end{cases} \tag{S19c}$$

thus, the term $\mathcal{H}^{(1)}_{sm,s'm'}$, defined in Eq. (S14), can be simplified to the following formula:

$$\mathcal{H}^{(1)}_{sm,s'm'} = \frac{2c\tilde{\delta}_{mm'}}{\pi\left(m^2 - (m')^2\right)}\sqrt{\frac{mm'}{n_s n_{s'}}}\mathbf{A}_{ss'}, \tag{S20}$$

where $\tilde{\delta}_{mm'}$ equals 1 if $m + m'$ is an odd number, and 0 otherwise. Using these simplifications, we obtain a new formula for the nonzero elements of the rotation matrix $\mathbf{R}_{sm,s'm'}$:

$$\mathbf{R}_{sm,s'm'} = -\frac{2L\tilde{\delta}_{mm'}\sqrt{mm'n_s n_{s'}}}{\pi^2\left(m^2 - (m')^2\right)(mn_{s'} - m'n_s)}\mathbf{A}_{ss'} \tag{S21}$$

This formula can be used only if the $\boldsymbol{E}_{sm}$ and $\boldsymbol{E}_{s'm'}$ states belong to different subsets of the basis, as explained in

Eq. (S13). In this case, the formula for the unnormalized Stokes polarization parameter takes the following form:

$$S_{i,\pm} = \sum_{s,s'} q^*_{\pm,s} q_{\pm,s'} \Bigg(\boldsymbol{\sigma}_{i,ss'} + \sum_{s''m''} \mathbf{R}^*_{s''m'',sm} \boldsymbol{\sigma}_{i,s''s'} + \mathbf{R}_{s''m'',s'm'} \boldsymbol{\sigma}_{i,ss''} \Bigg) \quad \text{(S22)}$$

where $\boldsymbol{\sigma} = (\boldsymbol{\sigma}_z, \boldsymbol{\sigma}_x, \boldsymbol{\sigma}_y)$ as the considered Hamiltonian in written in the linear basis. The $\mathbf{A}$ matrix is anti-Hermitian and symmetric, so $\mathbf{A}^*_{ss'} = -\mathbf{A}_{ss'} = -\mathbf{A}_{s's}$ and $\mathbf{R}^*_{sm,s'm'} = -\mathbf{R}_{sm,s'm'}$. To simplify the formula for the Stokes polarization parameter, we consider the rotated Pauli matrix defined as:

$$\tilde{\boldsymbol{\sigma}}_{i,ss'} = \boldsymbol{\sigma}_{i,ss'} + \sum_{s''m''} \mathbf{R}_{s''m'',s'm'} \boldsymbol{\sigma}_{i,ss''} - \mathbf{R}_{s''m'',sm} \boldsymbol{\sigma}_{i,s''s'} \quad \text{(S23)}$$

Substituting the formula from Eq. (S21) into Eq. (S23) gives:

$$\tilde{\boldsymbol{\sigma}}_{i,ss'} = \boldsymbol{\sigma}_{i,ss'} + \frac{2L}{\pi^2} \sum_{s''m''} \frac{\sqrt{m''mn_{s''}n_s}\mathbf{A}_{s''s}\tilde{\delta}_{m''m}}{\left((m'')^2 - m^2\right)(m''n_s - mn_{s''})} \boldsymbol{\sigma}_{i,s''s'} - + \frac{\sqrt{m''m'n_{s''}n_{s'}}\mathbf{A}_{s''s'}\tilde{\delta}_{m''m'}}{\left((m'')^2 - (m')^2\right)(m''n_{s'} - m'n_{s''})} \boldsymbol{\sigma}_{i,ss''} \quad \text{(S24)}$$

which can be further simplified for each Stokes polarization parameter. The $\boldsymbol{\sigma}_1$ matrix for the Stokes S_1 polarization parameter contains only a diagonal term, so we obtain:

$$\tilde{\boldsymbol{\sigma}}_{1,ss'} = \boldsymbol{\sigma}_{1,ss'} + \frac{2L}{\pi^2} \sum_{m''} \frac{\sqrt{m''mn_{s'}n_s}\mathbf{A}_{s's}\tilde{\delta}_{m''m}}{\left((m'')^2 - m^2\right)(m''n_s - mn_{s'})} \boldsymbol{\sigma}_{1,s's'} - + \frac{\sqrt{m''m'n_sn_{s'}}\mathbf{A}_{ss'}\tilde{\delta}_{m''m'}}{\left((m'')^2 - (m')^2\right)(m''n_{s'} - m'n_s)} \boldsymbol{\sigma}_{1,ss}. \quad \text{(S25)}$$

This can be further simplified to the following formula:

$$\tilde{\boldsymbol{\sigma}}_{i,ss'} = \tilde{\sigma}_{1,ss'} + \frac{2L\sqrt{n_sn_{s'}}\mathbf{A}_{ss'}}{\pi^2} \left(g(m, n_{s'})\boldsymbol{\sigma}_{1,s's'} - + g(m', n_s)\boldsymbol{\sigma}_{1,ss}\right), \quad \text{(S26)}$$

where the s and s' polarizations determine the values of m and m', so we set sm and $s'm'$, which correspond to $X(m+1)$ or $Y(m)$. The function $g(m,n)$ is a sum over all states present in the cavity and is defined as:

$$g(m,n) = \sum_{m'} \frac{\sqrt{m'm}\tilde{\delta}_{m'm}}{(m'^2 - m^2)(m'n_s - mn)} \quad \text{(S27)}$$

where n_s is the refractive index for s polarization, which is the same as for mode number m. In this case, the formula for the rotated first Pauli matrix is as follows:

$$\tilde{\boldsymbol{\sigma}}_1 = \begin{bmatrix} 1 & -\beta_1 \\ \beta_1 & -1 \end{bmatrix} \quad \text{(S28)}$$

where β_1 is given by:

$$\beta_1 = -i\frac{2L\varepsilon_{xz}\sqrt{n_Xn_Y}k_y}{\pi^2\varepsilon_{zz}} \left(g(m_Y, n_X) + + (g(m_X, n_Y)\right) \quad \text{(S29)}$$

Here, m_X and m_Y denote the mode numbers for horizontally and vertically polarized modes. The matrix $\tilde{\boldsymbol{\sigma}}_1$ is Hermitian because β_1 is purely imaginary. In this case, the S_1 Stokes polarization parameter, according to Eq. (S22) with $\boldsymbol{q}_\pm$ and $\tilde{\boldsymbol{\sigma}}_1$ defined in Eqs. (S18) and (S28), is given by:

$$S_{1,\pm} = \boldsymbol{q}^\dagger \tilde{\boldsymbol{\sigma}}_1 \boldsymbol{q} = = \begin{bmatrix} h_1 \pm \sqrt{h_1^2 + h_3^2} \\ ih_3 \end{bmatrix}^\dagger \begin{bmatrix} 1 & -\beta_1 \\ \beta_1 & 1 \end{bmatrix} \begin{bmatrix} h_1 \pm \sqrt{h_1^2 + h_3^2} \\ ih_3 \end{bmatrix} = 2(h_1 - i\beta_1h_3)\left(h_1 \pm \sqrt{h_1^2 + h_3^2}\right) \quad \text{(S30)}$$

In this case, since $h_1 \pm \sqrt{h_1^2 + h_3^2} \neq 0$, C-points can only occur if $h_1 - i\beta_1h_3 = 0$.

The same consideration applies to the S_2 and S_3 Stokes polarization parameters. In these cases, the Pauli matrices $\boldsymbol{\sigma}_2$ and $\boldsymbol{\sigma}_3$ have only anti-diagonal terms (see the comment below in Eq. (S22)), so we simplify the formula for the rotated Pauli matrices given in Eq. (S24) to the following formula:

$$\tilde{\boldsymbol{\sigma}}_{i,ss'} = \boldsymbol{\sigma}_{i,ss'} + \frac{2L}{\pi^2} \sum_{m''} \frac{\sqrt{m''mn_{\tilde{s}'}n_s}\mathbf{A}_{\tilde{s}'s}\tilde{\delta}_{m''m}}{\left((m'')^2 - m^2\right)(m''n_s - mn_{\tilde{s}'})} \boldsymbol{\sigma}_{i,\tilde{s}'s'} - + \frac{\sqrt{m''m'n_{\tilde{s}}n_{s'}}\mathbf{A}_{\tilde{s}s'}\tilde{\delta}_{m''m'}}{\left((m'')^2 - (m')^2\right)(m''n_{s'} - m'n_{\tilde{s}})} \boldsymbol{\sigma}_{i,s\tilde{s}} \quad \text{(S31)}$$

where $\tilde{s}$ is defined as:

$$\tilde{s} = \begin{cases} 1 & \text{if } s = 2 \\ 2 & \text{if } s = 1 \end{cases} \quad \text{(S32)}$$

The final formula for the rotated Pauli matrices $\tilde{\boldsymbol{\sigma}}_2$ and $\tilde{\boldsymbol{\sigma}}_3$ in this case is given by:

$$\tilde{\sigma}_{i,ss'} = \boldsymbol{\sigma}_{i,ss'} + \frac{2L}{\pi^2} \left(\sqrt{n_{\tilde{s}_2}n_{s_1}}\mathbf{A}_{\tilde{s}_2s_1} g(m_1, n_{\tilde{s}_2}) \boldsymbol{\sigma}_{i,\tilde{s}_2s_2} - \sqrt{n_{\tilde{s}_1}n_{s_2}}\mathbf{A}_{\tilde{s}_1s_2} g(m_2, n_{\tilde{s}_1}) \boldsymbol{\sigma}_{i,s_1\tilde{s}_1}\right) \quad \text{(S33)}$$

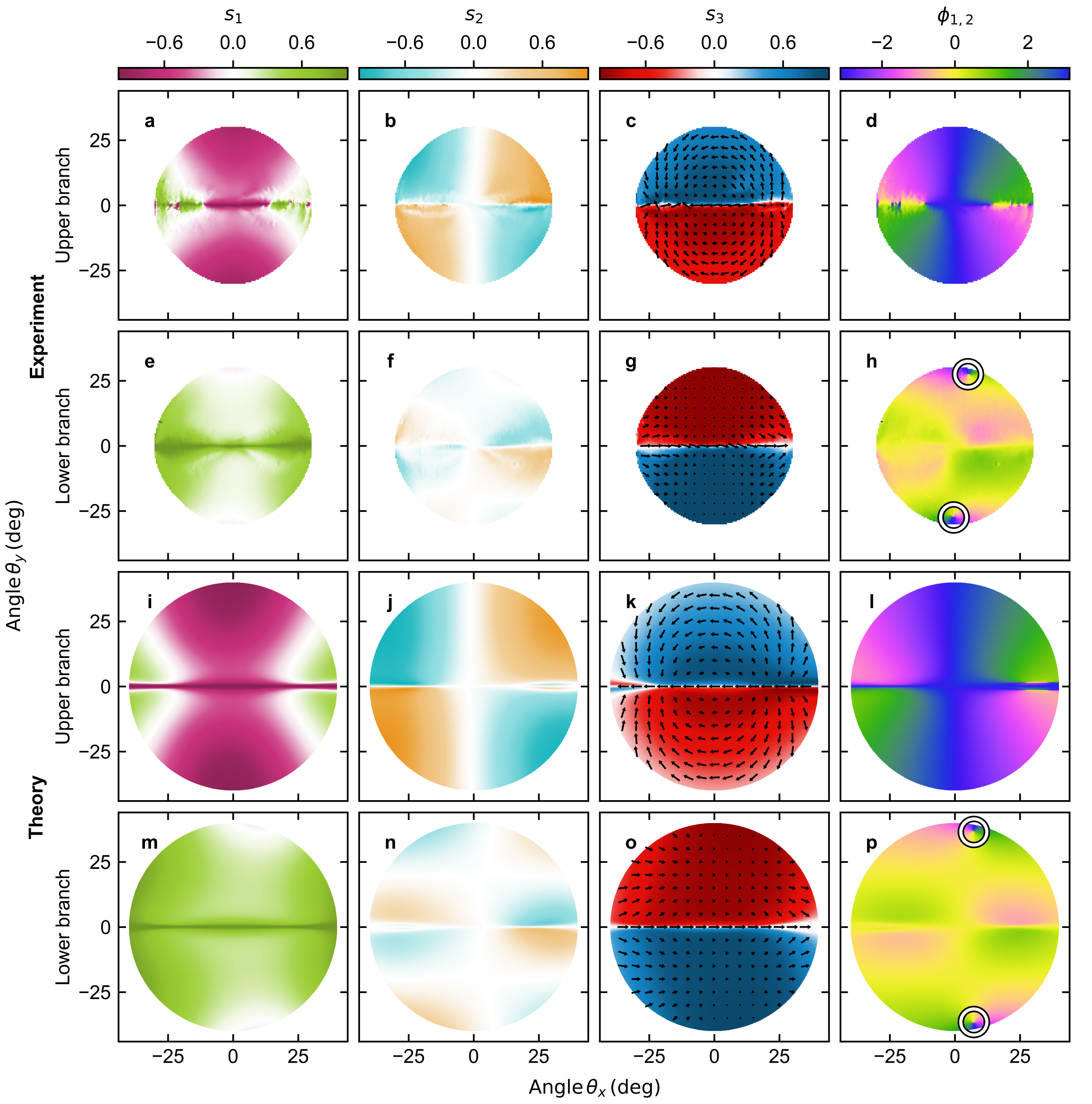

FIG. S9. Stokes parameters and phase in negative detuning. **a-h** Experimentally and **i-p** theoretically obtained Stokes parameters **a**, **e**, **i**, **m** s_1, **b**, **f**, **j**, **n**, s_2, **c**, **g**, **k**, **o** s_3, and **d**, **h**, **l**, **p** Stokes phase $\phi_{1,2} = \arg(s_1 + is_2)$ for **a-d, i-l** upper and **e-h, m-p** lower branch in the case of the positive detuning of $2\hbar\Delta = \hbar\mathrm{Re}\left(\omega_{H(m+1)} - \omega_{V(m)}\right) = -1.7\,\mathrm{meV}$. The black arrows on the s_3 map correspond to $\boldsymbol{s}_\parallel = [s_1, s_2]^T$. The white circles on the Stokes phase $\phi_{1,2}$ maps mark the position of the C-points.

where $g(m,n)$ is defined in Eq. (S27). Finally, the rotated matrices for the S_2 and S_3 Stokes polarization parameters are given by:

$$\tilde{\boldsymbol{\sigma}}_2 = \begin{bmatrix} 0 & 1+\beta_2 \\ 1-\beta_2 & 0 \end{bmatrix} \tag{S34a}$$

$$\tilde{\boldsymbol{\sigma}}_3 = \begin{bmatrix} \beta_3 g(m_X, n_Y) & -i-i\beta_2 \\ i-i\beta_2 & -\beta_3 g(m_Y, n_X) \end{bmatrix} \tag{S34b}$$

where:

$$\beta_2 = -i\frac{4L\varepsilon_{xz} n_X k_x}{\pi^2 \varepsilon_{zz}} g(m_X, n_X) \tag{S35a}$$

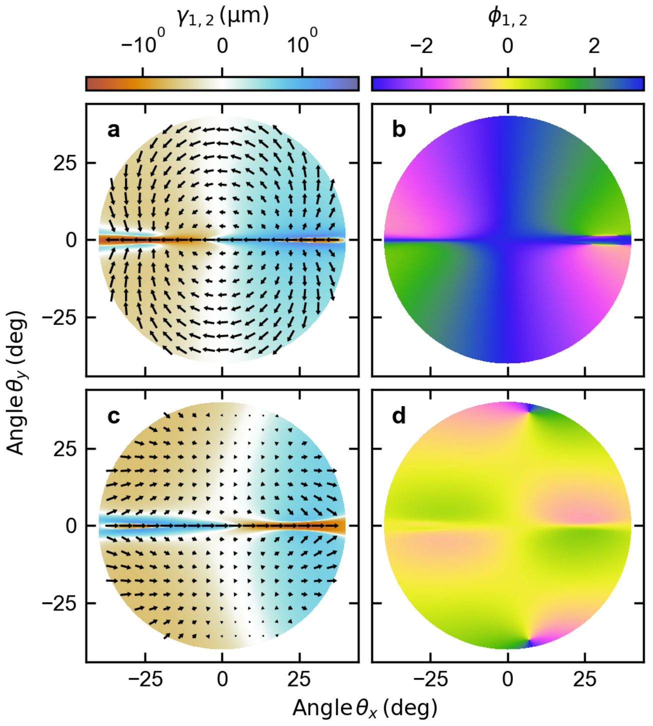

FIG. S10. Two dimensional divergence and Stokes phase for $\Delta = -1.7\,\mathrm{meV}$ for Sample B ($\theta = 33.5°$). **a** and **c** two dimensional divergence $\gamma_{1,2}$ for upper **a** and lower **c** branch, respectively. The arrows denote the $\boldsymbol{s}_\parallel$ vector. **b** and **d** Stokes phase for upper and lower branch, respectively.

$$\beta_3 = \frac{4L\varepsilon_{xz}\sqrt{n_X n_Y}k_y}{\pi^2\varepsilon_{zz}} \tag{S35b}$$

The matrices $\tilde{\boldsymbol{\sigma}}_2$ and $\tilde{\boldsymbol{\sigma}}_3$ are Hermitian operators because β_2 is purely imaginary and β_3 is purely real. Using the formulas presented in Eq. (S18) and Eq. (S22), we obtain the following relations for the S_2 and S_3 Stokes polarization parameters:

$$S_{2,\pm} = \boldsymbol{q}^\dagger\tilde{\boldsymbol{\sigma}}_2\boldsymbol{q} = \begin{bmatrix} h_1 \pm \sqrt{h_1^2+h_3^2} \\ ih_3 \end{bmatrix}^\dagger \cdot \begin{bmatrix} 0 & 1+\beta_2 \\ 1-\beta_2 & 0 \end{bmatrix} \begin{bmatrix} h_1 \pm \sqrt{h_1^2+h_3^2} \\ ih_3 \end{bmatrix} = 2i\beta_2 h_3\left(h_1 \pm \sqrt{h_1^2+h_3^2}\right) \tag{S36a}$$

$$S_{3,\pm} = \boldsymbol{q}^\dagger\tilde{\boldsymbol{\sigma}}_3\boldsymbol{q} = \begin{bmatrix} h_1 \pm \sqrt{h_1^2+h_3^2} \\ ih_3 \end{bmatrix}^\dagger \cdot \begin{bmatrix} \beta_3 g(m_X,n_Y) & -i-i\beta_2 \\ i-i\beta_2 & -\beta_3 g(m_Y,n_X) \end{bmatrix} \begin{bmatrix} h_1 \pm \sqrt{h_1^2+h_3^2} \\ ih_3 \end{bmatrix} = \beta_3\left[\left(h_1 \pm \sqrt{h_1^2+h_3^2}\right)^2 g(m_X,n_Y) - h_3^2 g(m_Y,n_X)\right] + 2h_3\left(h_1 \pm \sqrt{h_1^2+h_3^2}\right) \tag{S36b}$$

In the Hermitian limit, $\tilde{\boldsymbol{\sigma}}_2 \neq \boldsymbol{\sigma}_2$ if $k_x \neq 0$, and the $\boldsymbol{q}_\pm$ vectors are orthogonal. Thus, the S_2 Stokes polarization parameter is zero at $k_x = 0$ or $k_y = 0$, but is non-zero in other directions due to the non-zero values of β_2 and h_3 (see Eq. (S36a)). This indicates that the C-points can occur only in the $k_x = 0$ direction, since along the $k_y = 0$ direction we have $h_3 = 0$, making the S_3 Stokes polarization parameter zero.

According to the formula presented in Eq. (S30), the S_1 Stokes polarization parameter is zero if $h_1 - i\beta_1 h_3 = 0$. Additionally, the S_2 Stokes polarization parameter is zero at $k_x = 0$. Therefore, using the formulas for h_1 and h_3 defined in Eq. (S17) and β_1 defined in Eq. (S29), we obtain the following relation for the position of the C-points in k-space:

$$k_{y,CP} = \pm\sqrt{\frac{\Delta}{\Sigma(m_Y)\frac{4Lc\sqrt{(m+1)m}}{(2m+1)\pi^3}\left(\frac{\varepsilon_{xz}}{\varepsilon_{zz}}\right)^2 - \delta_y}} \tag{S37}$$

where $\Sigma(m) = g(m_Y,n_X) + g(m_Y+1,n_Y)$. The sign of δ_y in the Rashba-Dresselhaus coupling depends on the cavity thickness (it is negative for small thickness and positive for a thick cavity). Since $\Sigma(m_Y)$ is always positive, C-points can occur only for positive detuning ($\Delta > 0$).

C. Parameters of simulation

In this approach, we use the following values for all parameters. The dielectric tensor $\hat{\varepsilon}$ is the same as in Eq. (S4a) with $n_o = 1.5$ and $n_e = 1.7$. Cavity thickness is equal to $L = 2120\,\mathrm{nm}$. The cavity photon lifetime is determined by the value of ζ_s [see Eq. (S2)], which is equal to $\zeta_X = 82\,\mu\mathrm{m}^{-1}$ and $\zeta_Y = 78\,\mu\mathrm{m}^{-1}$ for horizontally and vertically polarized mode, respectively. We performed simulation for 13-th horizontal and 12-th vertical mode in a cavity. Using the perturbation theory we take into account the interaction with modes with mode numbers from 5 to 20. The rotation angle of molecule $\theta = 1.02\theta_r$ and $\theta = 0.98\theta_r$ for positive and negative detuning, respectively, where θ_r denotes the angle for which the $\Delta = 0$. The refractive index outside cavity is equal $n_a = 1$.

SIII. TRANSFER MATRIX METHOD

A. General information

In our approach we used the standard transfer matrix method presented in Berreman's [3] and Schubert's [4] papers. In this approach the relation between the two-dimensional vector (in $x-y$ plane, which is the same as the plane of the cavity) of electric $\boldsymbol{E}(z)$ and magnetic

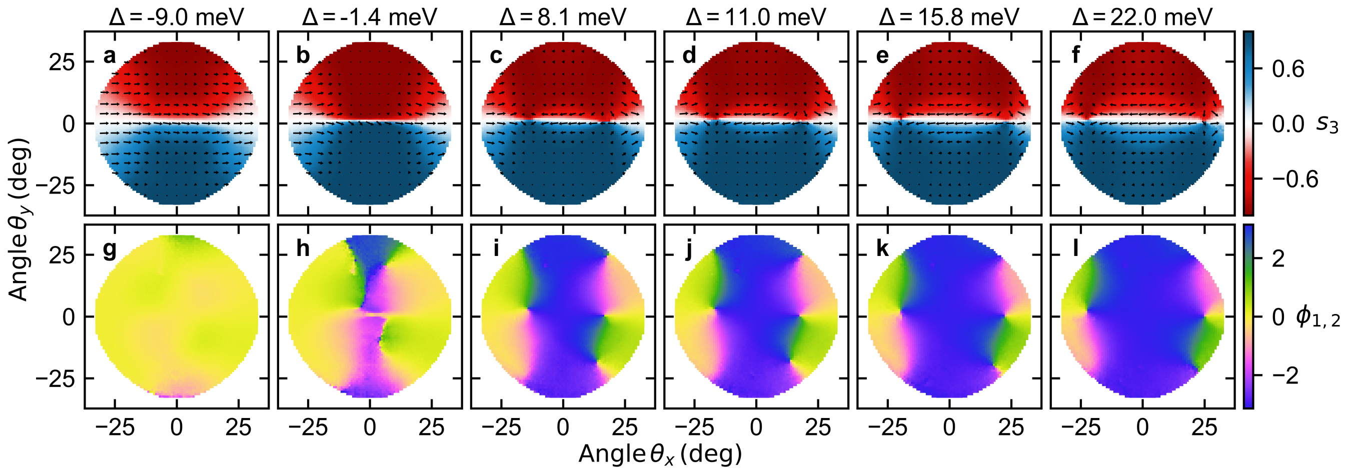

FIG. S11. Tunability of the polarization singularities. **a-f** Lower branch s_3 Stokes parameter for as a function of detuning with overlaid $\boldsymbol{s}_{\parallel} = [s_1, s_2]^T$ as black arrows. **g-i** Corresponding Stokes phase $\phi_{1,2} = \arg(s_1 + is_2)$ showing changing position of the singularities in reciprocal space.

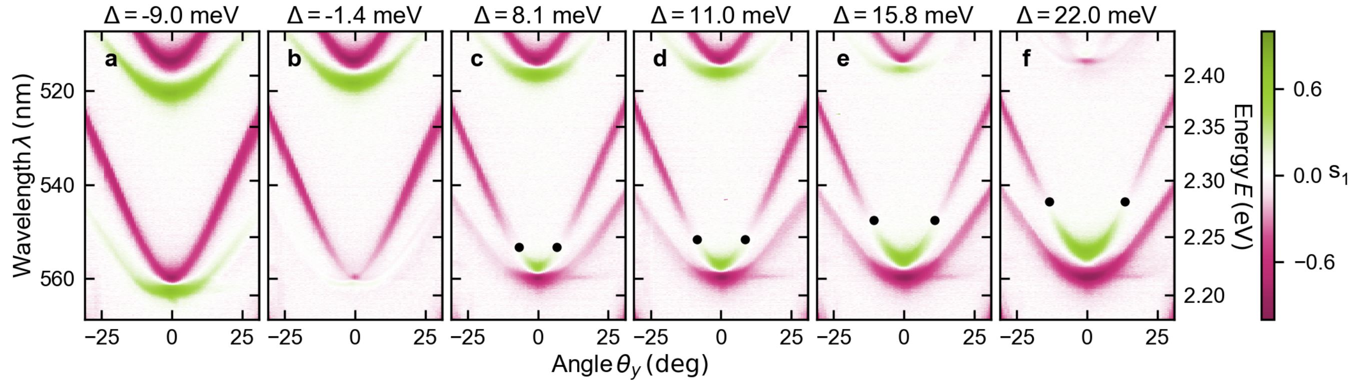

FIG. S12. s_1 Stokes polarization parameters for different value of detuning. The black dots at panels **c-f** mark position of C-points. For the negative **a** and close to zero **b** detuning the C-points are not observed.

$\boldsymbol{H}(z)$ field is given by the following equation:

$$\partial_z \begin{bmatrix} \boldsymbol{E}(z) \\ \boldsymbol{H}(z) \end{bmatrix} = = \begin{bmatrix} \mathbf{S}_{11} & \mathbf{S}_{12} \\ \mathbf{S}_{21} & \mathbf{S}_{22} \end{bmatrix} \begin{bmatrix} \boldsymbol{E}(z) \\ \boldsymbol{H}(z) \end{bmatrix} = \mathbf{T}(z) \begin{bmatrix} \boldsymbol{E}(z) \\ \boldsymbol{H}(z) \end{bmatrix} \tag{S38}$$

where the matrices $\mathbf{S}_{ij}$ depend on z-cooridinate and have following form:

$$\mathbf{S}_{11} = -\frac{ik_0}{\varepsilon_{zz}} \begin{bmatrix} \kappa_x \varepsilon_{zx} & \kappa_x \varepsilon_{zy} \\ \kappa_y \varepsilon_{zx} & \kappa_y \varepsilon_{zy} \end{bmatrix} \tag{S39a}$$

$$\mathbf{S}_{12} = \frac{ik_0\eta_0}{\varepsilon_{zz}} \begin{bmatrix} \kappa_x\kappa_y & \varepsilon_{zz} - \kappa_x^2 \\ \kappa_y^2 - \varepsilon_{zz} & -\kappa_x\kappa_y \end{bmatrix} \tag{S39b}$$

$$\mathbf{S}_{21} = \frac{ik_0}{\eta_0} \begin{bmatrix} -\tilde{\varepsilon}_{yx} - \kappa_x\kappa_y & -\tilde{\varepsilon}_{yy} + \kappa_x^2 \\ \tilde{\varepsilon}_{xx} - \kappa_y^2 & \tilde{\varepsilon}_{xy} + \kappa_x\kappa_y \end{bmatrix} \tag{S39c}$$

$$\mathbf{S}_{22} = \frac{ik_0}{\varepsilon_{zz}} \begin{bmatrix} -\kappa_y \varepsilon_{yz} & \kappa_x \varepsilon_{yz} \\ \kappa_y \varepsilon_{xz} & -\kappa_x \varepsilon_{xz} \end{bmatrix}. \tag{S39d}$$

Here $\eta_0 = \sqrt{\mu_0/\varepsilon_0}$ denotes the standard vacuum impedance, ε_{ij} is the element of dielectric tensor, $\tilde{\varepsilon}_{ij} = \varepsilon_{ij} - \varepsilon_{iz}\varepsilon_{zj}/\varepsilon_{zz}$, $k_0 = 2\pi/\lambda_0$ (wavewvector in vacuum), $\kappa_x = n_a \sin(\theta)\cos(\varphi)$ and $\kappa_y = n_a \sin(\theta)\sin(\varphi)$, where n_a is a refractive index outside the structure (in this case it is the refractive index for air, so $n_a = 1$), θ denotes the angle of incident wave and φ is the azimuthal angle of the cavity plane. The transfer matrix for a full cavity is a product of the transfer matrices for all layers presented in the cavity, so:

$$\begin{bmatrix} \boldsymbol{E}(z_0) \\ \boldsymbol{H}(z_0) \end{bmatrix} = \mathbf{T}_f \begin{bmatrix} \boldsymbol{E}(z_N) \\ \boldsymbol{H}(z_N) \end{bmatrix} \tag{S40a}$$

$$\mathbf{T}_f = \prod_{i=1}^{N} \exp\left(\mathbf{T}(z_i)(z_{i-1} - z_i)\right) \tag{S40b}$$

In standard approach the amplitude of incident $\boldsymbol{A} = [A_{TM}, A_{TE}]^T$, reflected $\boldsymbol{B} = [B_{TM}, B_{TE}]^T$ and transmitted $\boldsymbol{C} = [C_{TM}, C_{TE}]^T$ is given in the $TE - TM$ basis, so we introduce the matrices $\mathbf{L}_a$ and $\mathbf{L}_f$ which transform the amplitude from $TE - TM$ basis to $x - y$ basis. These matrices are equal to:

$$\mathbf{L}_a = \begin{bmatrix} -\cos(\theta)\cos(\varphi) & \sin(\varphi) & \cos(\theta)\cos(\varphi) & \sin(\varphi) \\ -\cos(\theta)\sin(\varphi) & -\cos(\varphi) & \cos(\theta)\sin(\varphi) & -\cos(\varphi) \\ \frac{n_a}{\eta_0}\sin(\varphi) & \frac{n_a}{\eta_0}\cos(\theta)\cos(\varphi) & \frac{n_a}{\eta_0}\sin(\varphi) & -\frac{n_a}{\eta_0}\cos(\theta)\cos(\varphi) \\ -\frac{n_a}{\eta_0}\cos(\varphi) & \frac{n_a}{\eta_0}\cos(\theta)\sin(\varphi) & -\frac{n_a}{\eta_0}\cos(\varphi) & -\frac{n_a}{\eta_0}\cos(\theta)\sin(\varphi) \end{bmatrix} \tag{S41}$$

$$\mathbf{L}_f = \begin{bmatrix} -\cos(\vartheta)\cos(\varphi) & \sin(\varphi) & 0 & 0 \\ -\cos(\vartheta)\sin(\varphi) & -\cos(\varphi) & 0 & 0 \\ \frac{n_f}{\eta_0}\sin(\varphi) & \frac{n_f}{\eta_0}\cos(\vartheta)\cos(\varphi) & 0 & 0 \\ -\frac{n_f}{\eta_0}\cos(\varphi) & \frac{n_f}{\eta_0}\cos(\vartheta)\sin(\varphi) & 0 & 0 \end{bmatrix} \tag{S42}$$

where n_f denotes the refractive index of the isotropic medium at the right side of the sample (air, in our case, so $n_f = 1$) and $\vartheta = \arcsin(n_a \sin(\theta)/n_f)$. The final relation between the amplitudes of incident, reflected, and transmitted waves in $TE-TM$ basis is given by following equation:

$$\begin{bmatrix} \boldsymbol{A} \\ \boldsymbol{B} \end{bmatrix} = \mathbf{L}_a^{-1}\mathbf{T}_f\mathbf{L}_f \begin{bmatrix} \boldsymbol{C} \\ \mathbf{0} \end{bmatrix} = \begin{bmatrix} \mathbf{T}_{11} & \mathbf{T}_{12} \\ \mathbf{T}_{21} & \mathbf{T}_{22} \end{bmatrix} \begin{bmatrix} \boldsymbol{C} \\ \mathbf{0} \end{bmatrix} \tag{S43}$$

where $\mathbf{T}_{ij}$ denotes the 2×2 submatrices of full transfer matrix between amplitude in $TE - TM$ basis. In such a way (scattering geometry), the amplitudes of reflected and transmitted waves are equal to:

$$\boldsymbol{B} = \mathbf{T}_{21}\mathbf{T}_{11}^{-1}\mathbf{A} \tag{S44a}$$

$$\boldsymbol{C} = \mathbf{T}_{11}^{-1}\boldsymbol{A} \tag{S44b}$$

In scattering geometry some effects for example exceptional points (degeneracy points in reciprocal space) cannot be observed in simulation results, so we used another approach, which is called outgoing wave boundary conditions, for which the amplitude of an incident wave is equal to zero. In this approach, the Eq. (S43) can be rewritten to the following formula:

$$\begin{bmatrix} \mathbf{T}_{11} & \mathbf{0} \\ \mathbf{T}_{21} & -\boldsymbol{\sigma}_0 \end{bmatrix} \begin{bmatrix} \boldsymbol{C} \\ \boldsymbol{B} \end{bmatrix} = \begin{bmatrix} \mathbf{0} \\ \mathbf{0} \end{bmatrix} \tag{S45}$$

where $\boldsymbol{\sigma}_0$ denotes the 2×2 identity matrix. A nontrivial solution ($\boldsymbol{B} \neq \boldsymbol{C} \neq \mathbf{0}$) exists if $\det(\mathbf{T}_{11}) = 0$, which is satisfied only for complex energy values $E = E_r - i\Gamma$. Here, E_r and Γ represent the resonant energy and the lifetime of the photon in the cavity, respectively. The complex solution was found, by using the standard Newton algorithm for complex root, with the initial value calculated as a quadratic extrapolation based on the three previous values.

The coefficient $\boldsymbol{A}$, $\boldsymbol{B}$, and $\boldsymbol{C}$ is given in the $TE - TM$ basis and it yields the normalized Stokes polarization parameters in $TE - TM$ basis, which are equal to:

$$\tilde{s}_0 = |v_{TM}|^2 + |v_{TE}|^2 \tag{S46a}$$

$$\tilde{s}_1 = |v_{TM}|^2 - |v_{TE}|^2 \tag{S46b}$$

$$\tilde{s}_2 = v_{TM}^* v_{TE} + v_{TE}^* v_{TM} \tag{S46c}$$

$$\tilde{s}_3 = i\left(v_{TE}^* v_{TM} - v_{TM}^* v_{TE}\right) \tag{S46d}$$

where $\boldsymbol{v}$ denotes the $\boldsymbol{A}$, $\boldsymbol{B}$ or $\boldsymbol{C}$ amplitudes. In the scattering geometry, the incident wave is normalized, so $|\boldsymbol{A}| = 1$. In the outgoing wave geometry, $|\boldsymbol{A}| = 0$, so we normalize the $\boldsymbol{B}$ and $\boldsymbol{C}$ amplitudes such that $|\boldsymbol{B}| = |\boldsymbol{C}| = 1$. In this experiment, the intensity of light for certain polarization was collected in the $x - y$ basis, so the normalized Stokes polarization parameters in this basis are given by:

$$s_0 = |u_x|^2 + |u_y|^2 \tag{S47a}$$

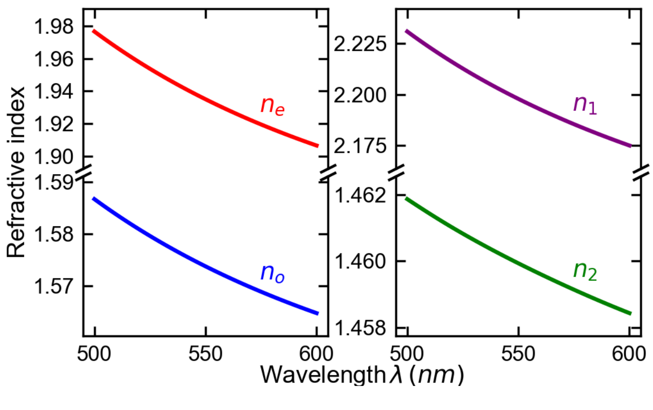

FIG. S13. Dispersion relation of refractive indices for liquid crystal used in sample A – blue and red curves denote the ordinary and extraordinary refractive indices, respectively. Dispersion relation of refractive indices for materSiO$_2$ and purple TiO$_2$.

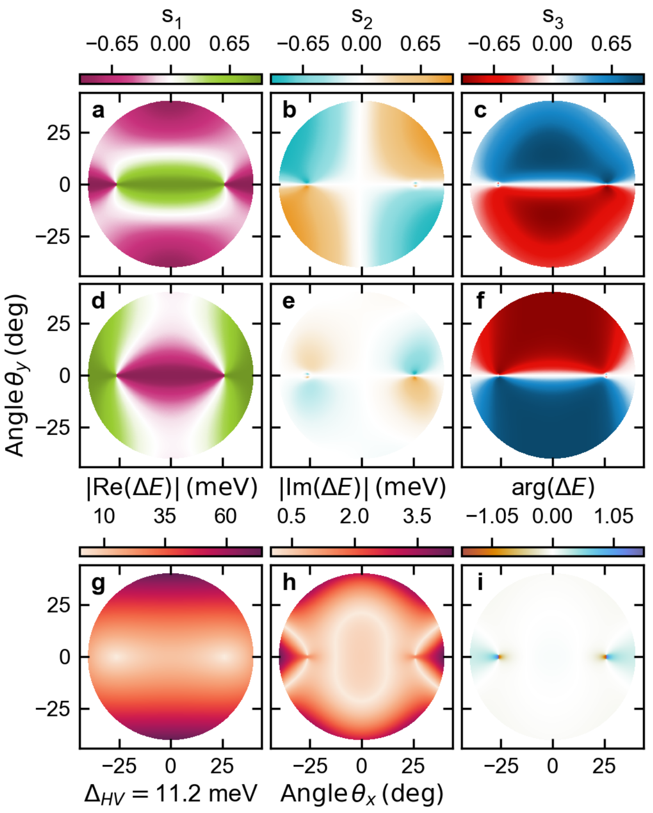

FIG. S14. Polarization patterns and difference between energies for two branches for Sample A with 4 pairs of layers in DBRs. **a-c** and **d-f** present polarization patterns for upper and lower branches, respectively. **g** and **h** presents the absolute value of the real and imaginary part difference between energies for two branches, respectively. **i** presents the argument of complex number – difference between energies for both branches. The Δ_{HV} denote the difference between energies for two modes for perpendicular incident wave.

$$s_1 = |u_x|^2 - |u_y|^2 \tag{S47b}$$

$$s_2 = u_x^* u_y + u_y^* u_x \tag{S47c}$$

$$s_3 = i\left(u_y^* u_x - u_x^* u_y\right) \tag{S47d}$$

where $\boldsymbol{u}$ denotes the amplitude of incident, reflected or transmitted wave in $x-y$ basis. The relation between the amplitudes in $TE-TM$ basis and $x-y$, which is given by:

$$\begin{bmatrix} u_x \\ u_y \end{bmatrix} = \begin{bmatrix} -\cos(\theta_1)\cos(\varphi) & \sin(\varphi) \\ -\cos(\theta_1)\sin(\varphi) & -\cos(\varphi) \end{bmatrix} \begin{bmatrix} v_{TM} \\ v_{TE} \end{bmatrix} \tag{S48}$$

where $\theta_1 = \theta$ for positive direction and $\theta_1 = \pi - \theta$ for negative direction of propagation. Using the relation between these two bases, we find the relation between Stokes polarization patterns in the $TE-TM$ basis Eq. (S46) and $x-y$ basis Eq. (S47), which has the following form for $\theta = 0$ (parallel beam for polarization setup):

$$\begin{bmatrix} s_0 \\ s_1 \\ s_2 \\ s_3 \end{bmatrix} = \begin{bmatrix} 1 & 0 & 0 & 0 \\ 0 & \cos(2\varphi) & \mp\sin(2\varphi) & 0 \\ 0 & \pm\sin(2\varphi) & \cos(2\varphi) & 0 \\ 0 & 0 & 0 & 1 \end{bmatrix} \begin{bmatrix} \tilde{s}_0 \\ \tilde{s}_1 \\ \tilde{s}_2 \\ \tilde{s}_3 \end{bmatrix} \tag{S49}$$

where the upper sign is for a wave that propagates on the right side (positive z) and the lower sign is for the wave that propagates on the left side (negative z).

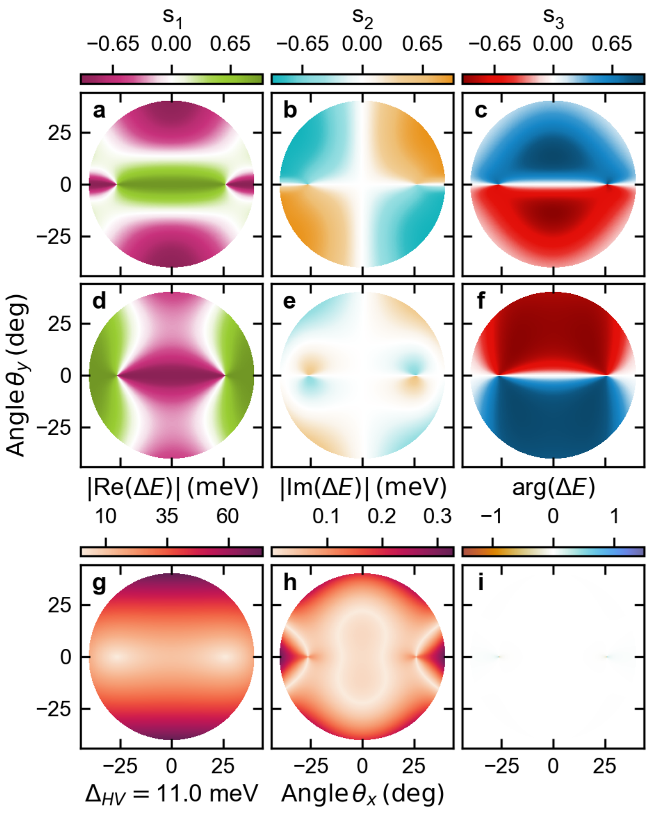

FIG. S15. Polarization patterns and difference between energies for two branches for Sample A with 8 pairs of layers in DBRs. **a-c** and **d-f** present polarization patterns for upper and lower branches, respectively. **g** and **h** presents the absolute value of the real and imaginary part difference between energies for two branches, respectively. **i** presents the argument of complex number – difference between energies for both branches. The Δ_{HV} denote the difference between energies for two modes for perpendicular incident wave.

B. Parameters of the sample

1. *Sample A*

Sample A consists of two Distributed Bragg Reflectors (DBRs) centered at 550 nm, made from 6 pairs of TiO_2/SiO_2 layers, with SiO_2 as the top layer. The dispersion relations of refractive index for TiO_2 and SiO_2

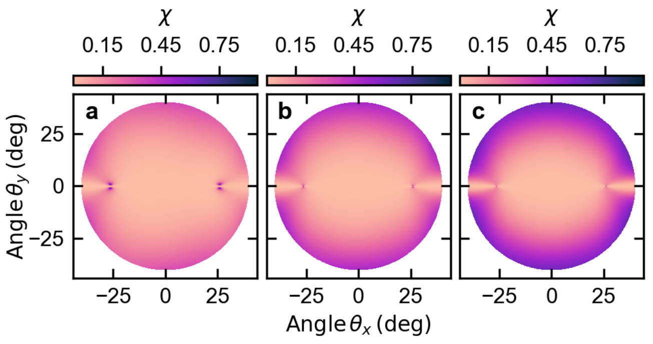

FIG. S16. Value of non-othogonality χ in a function of number of pairs of layers in DBRs for Sample A. **a** – 4 pairs, **b** – 6 pairs, **c** – 8 pairs. The rest parameters are the same as for Sample A.

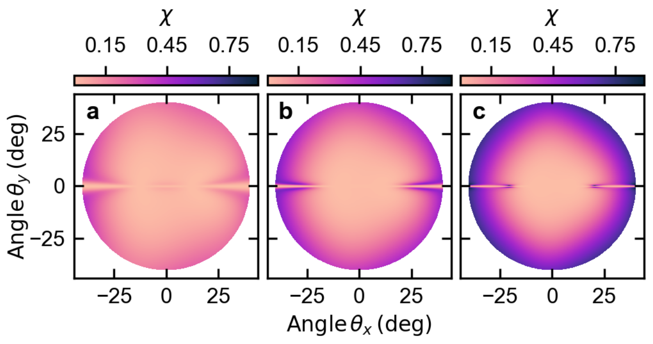

FIG. S17. Value of non-othogonality χ in a function of number of pairs of layers in DBRs for Sample B. **a** – 4 pairs, **b** – 6 pairs, **c** – 8 pairs. The rest parameters are the same as for Sample B.

layers are presented in Fig. S13. In this calculation, we assumed that the cavity consists of three layers between two DBRs. The first and third layers are the same and isotropic, with thickness 50 nm and refractive index $n = 1.5$. The second layer is a liquid crystal layer with n_o and n_e, which depends on the wavelength, and this dependence is presented in Fig. S13. The thickness of this layer is equal to 1163 nm. Dielectric tensor for different angles of liquid crystal molecule is defined in the same way as in Eq. (S4a). The angle of rotation of the molecule is equal to $\theta = 31.4°$. The comparison between experimental data collected for this sample and numerical data was presented in Fig. S18.

2. *Sample B*

Sample B consists of two Distributed Bragg Reflectors (DBRs) centered at 530 nm, made from 6 pairs of TiO_2/SiO_2 layers, with SiO_2 as the top layer. The dispersion relations of refractive index for TiO_2 and SiO_2 layers are presented in Fig. S13. In this calculation, we assumed that the cavity consists of three layers between two DBRs. The first and third layers are the same and isotropic, with thickness 30 nm and refractive index $n = 1.5$. The second layer is a liquid crystal layer with $n_e = 1.7114$ and $n_o = 1.5190$, which are independent of wavelength. The thickness of this layer is equal to 2385 nm. The angle of rotation of the molecule in the simulation is equal to $\theta = 33.5°$. The comparison between experimental data collected for this sample and numerical data was presented in Fig. S19.

SIV. ORIGIN OF ADDITIONAL C-POINTS AT LOWER BRANCH

To better understand the origin of the additional two C-points at the lower branch, we performed numerical calculations using the transfer matrix method for various detuning values and cavity geometries.

All the movies present the distribution of Stokes polarization parameters $s_{i,\pm}$ for the upper (**a-c**) and lower (**d-f**) branches, respectively. The arrows in these panels indicate the two-dimensional Stokes vector, defined as $\boldsymbol{s}_i = [s_j, s_k]$, where j and k correspond to the subscripts in the Stokes phase ϕ_{jk}. The Stokes phases ϕ_{ij} for the upper and lower branches are shown in **g-i** and **j-l**, respectively. The detuning was varied from approximately -7 meV to 24 meV in steps of about 0.1 meV.

- The "Movie for 1st cavity schematically presented in Fig. S4**ag**" presents results for a cavity similar to Sample A, assuming that the refractive indices of all layers are independent of wavelength. The schematic arrangement of layers in one DBR is shown in Fig. S4**ag**.
- The "Movie for 2nd cavity schematically presented in Fig. S4**an**" movie shows results for a similar cavity, but with an inverted ordering of layers in the DBRs. The schematic arrangement of layers in one DBR is shown in Fig. S4**an**.
- The "Movie for 3rd cavity schematically presented in Fig. S4**au**" presents results for a cavity similar to the first one, but with the number of layer pairs in the DBRs reduced from 6 to 4. The schematic arrangement of layers in one DBR is shown in Fig. S4**au**.
- The "Movie for 4th cavity schematically presented in Fig. S4**bb**" shows results for a cavity similar to the second one, but with the number of layer pairs in the DBRs increased from 6 to 8. The schematic arrangement of layers in one DBR is shown in Fig. S4**bb**.

Figure S4 shows the change of Stokes phase ϕ_{ij} as a function of detuning for these four types of cavities. The additional two C-points at the lower branch are only absent in the cavity with six or fewer pairs of layers in the DBRs, with an inverted layer ordering (see Figure S4**ah-am**). In all other cases (the investigated cavity

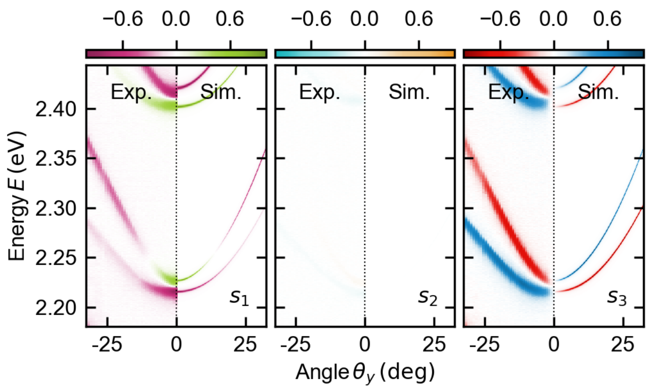

FIG. S18. Comparison of the dispersion relation between the experimental data (Exp.) and numerical data (Sim.) for the first sample. The s_i denotes the Stokes polarization parameter. The opposite colour for s_3 parameter is due to the opposite sign of angle for experiment and theory.

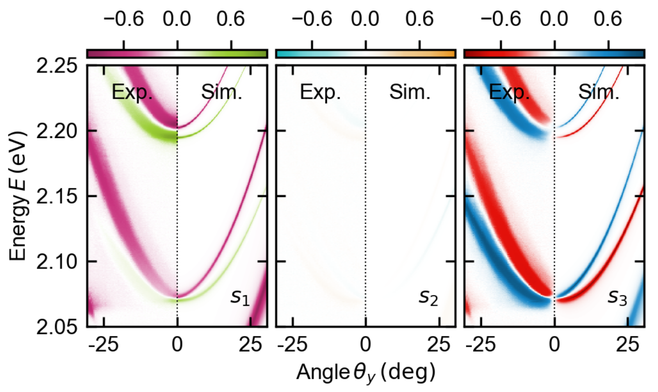

FIG. S19. Comparison of the dispersion relation between the experimental data (Exp.) and numerical data (Sim.) for the second sample. The s_i denotes the Stokes polarization parameter. The opposite colour for s_3 parameter is due to the opposite sign of angle for experiment and theory.

[see Figure S4**aa-af**], the cavity with four pairs of DBR layers in normal order [see Figure S4**ao-at**], and the cavity with eight pairs of DBR layers in inverted order [see Figure S4**av-ba**]), two C-points are observed for negative detuning and four C-points for positive detuning at the lower branch.

These results suggest that the occurrence of the additional C-points is linked to interactions with other modes in the cavity. This effect is driven by two key components. The first arises from the overlap between the electric fields of the cavity modes. The second is related to the discontinuity in the derivative of the refractive index, known as TE/TM splitting, which is typical of standard DBR cavities. However, in this case, due to the significantly larger relative difference between the high and low refractive indices, the effect is much more pronounced.

Furthermore, in a standard DBR cavity, horizontally and vertically polarized modes at resonance have identical electric field distributions for a perpendicularly incident wave. In contrast, in our case, these distributions differ because one mode is even and the other is odd. Hence both factors may contribute to the changes of the polarization-distribution, which become more pronounced as the number of DBR layers increases due to the greater number of discontinuity points and due to the incease of the volume where the electric fields of the modes overlap.

Nevertheless, all these effects arising from the interaction between the considered modes and other modes in the cavity can be incorporated as complex higher-order terms in the wavevector into the Hamiltonian presented in Eq. (2) in main manuscript.

SV. MERON STRUCTURE

All types of meron structures can be described using the general expression for the Stokes polarization parameter in polar coordinates, which is given by:

$$\boldsymbol{s} = \begin{bmatrix} \cos\left(\Phi\left(\varphi\right)\right)\sin\left(\Theta\left(r\right)\right) \\ \sin\left(\Phi\left(\varphi\right)\right)\sin\left(\Theta\left(r\right)\right) \\ \cos\left(\Theta\left(r\right)\right) \end{bmatrix} \tag{S50}$$

where $\Phi\left(\varphi\right) = v\varphi + \gamma$, with v representing the vorticity and γ representing the helicity, $\Theta\left(r\right)$ is a function specific to each meron structure. The Stokes polarization parameters for Bloch (Fig. 3**a** in the main text) and Néel (Fig. 3**b** in the main text) merons are obtained with the following values: $v = 1$, $\Theta\left(r\right) = \pi r/2r_{max}$, where r_{max} is the maximum radius, $\gamma = \pi/2$ for the Bloch meron, and $\gamma = 0$ for the Néel meron. The Stokes polarization parameters for the Node anti-meron (Fig. 3**c** in the main text) are obtained with the following values: $v = -1$, $\gamma = -\pi$, and $\Theta\left(x, y\right) = \pi/2 - \pi\mathrm{sgn}\left(y\right)\sqrt{x^2 + y^2}/2r_{max}$, where $\mathrm{sgn}\left(x\right)$ denotes the sign of x and $r_{max} = \sqrt{x_{max}^2 + y_{max}^2}$ and $x_{max} = y_{max}$.

SVI. RESULTS FOR NEGATIVE DETUNING

For negatively detuned H,V cavity modes we expect the C-points to vanish in accordance with our 2-mode Hamiltonian theory Eq. (2) in main text, discussed around Fig. 1 and 2 in the main text. In order to keep the negatively detuned cavity system as similar as possible in regards to previous results we used another sample for this regime (referred to as Sample B) with a different liquid crystal mixture. The results of angle-resolved tomography for a relatively low negative detuning value of $2\hbar\Delta = -1.7\,\mathrm{meV}$ (with a linewidth of approximately $3\,\mathrm{meV}$) for both upper and lower branches are presented in Figs. S9**a-d** and S9**e-h**. The results confirm the absence of C-points in the upper branch, while revealing the presence of two lemon-type $w = 1/2$ C-points in the lower branch at high angles $\boldsymbol{\theta} = [5^\circ, 28^\circ]^\mathrm{T}$ and $\boldsymbol{\theta} = [0^\circ, -28^\circ]^\mathrm{T}$

with a polarization distribution similar to a Bloch meron (see Fig. S10). Corresponding Berreman and Schubert simulations are shown in Figures S9**i-l** and S9**m-p** with good agreement. Comparison between experimental and theoretical dispersion relations for this sample is illustrated in Fig. S19.

[1] P. Oliwa, W. Bardyszewski, and J. Szczytko, Quantum mechanical-like approach with non-Hermitian effective Hamiltonians in spin-orbit coupled optical cavities, Phys. Rev. Res. **6**, 013324 (2024).
[2] K. Rechcińska, M. Król, R. Mazur, P. Morawiak, R. Mirek, K. Łempicka, W. Bardyszewski, M. Matuszewski, P. Kula, W. Piecek, P. G. Lagoudakis, B. Piętka, and J. Szczytko, Engineering spin-orbit synthetic Hamiltonians in liquid-crystal optical cavities, Science **366**, 727 (2019).
[3] D. W. Berreman, Optics in stratified and anisotropic media: 4×4-matrix formulation, J. Opt. Soc. Am. **62**, 502 (1972).
[4] M. Schubert, Polarization-dependent optical parameters of arbitrarily anisotropic homogeneous layered systems, Phys. Rev. B **53**, 4265 (1996).
